# Epigenetic inactivation of tumour suppressor coding and non-coding genes in human cancer: an update

**DOI:** 10.1098/rsob.170152

**Published:** 2017-09-20

**Authors:** Pere Llinàs-Arias, Manel Esteller

**Affiliations:** 1 Cancer Epigenetics Group, Cancer Epigenetics and Biology Program (PEBC), Bellvitge Biomedical Research Institute (IDIBELL), Barcelona, Catalonia, Spain; 2 Physiological Sciences Department, School of Medicine and Health Sciences, University of Barcelona (UB), Carrer de la Feixa Llarga, s/n, 08908 L'Hospitalet, Barcelona, Catalonia, Spain; 3 Institució Catalana de Recerca i Estudis Avançats (ICREA), Barcelona, Catalonia, Spain

**Keywords:** epigenetics, inactivation, methylation, cancer

## Abstract

Cancer cells undergo many different alterations during their transformation, including genetic and epigenetic events. The controlled division of healthy cells can be impaired through the downregulation of tumour suppressor genes. Here, we provide an update of the mechanisms in which epigenetically altered coding and non-coding tumour suppressor genes are implicated. We will highlight the importance of epigenetics in the different molecular pathways that lead to enhanced and unlimited capacity of division, genomic instability, metabolic shift, acquisition of mesenchymal features that lead to metastasis, and tumour plasticity. We will briefly describe these pathways, focusing especially on genes whose epigenetic inactivation through DNA methylation has been recently described, as well as on those that are well established as being epigenetically silenced in cancer. A brief perspective of current clinical therapeutic approaches that can revert epigenetic inactivation of non-coding tumour suppressor genes will also be given.

## 1. Introduction

Cell division is the molecular mechanism that allows us to grow, adapt and recover from stress. Essentially, it is a controlled process that keeps us alive. Tumour cells are the result of distortion of these mechanisms. They can grow faster and adapt better, living at our expense. They are an improved version of ourselves [1]. Cancer cells result from a set of aberrant alterations of DNA that lead to uncontrolled cell division. For many years the nature of these alterations has been studied. In 1969, Todaro and co-workers demonstrated that the administration of viral DNA and RNA had the ability to generate tumours [2,3]. This finding led to the discovery of the oncogenes, in other words, genes involved in growth and proliferation, which when deregulated contribute to malignant transformation. Two years later, Knudson identified another type of cancer-related gene through his retinoblastoma cancer studies, the tumour suppressor genes [4]. The function of this class of gene was none other than counteracting oncogenes, inhibiting growth in the absence of stimuli. A decade later, cancer studies met epigenetics. Epigenetics was first defined by Waddington in 1939 as ‘the causal interactions between genes and their products, which bring the phenotype into being’ [5]. Later the term was used to describe the occurrence of heritable changes in gene expression for which the DNA sequence is not altered [6]. One of the ways in which epigenetics is manifested is through DNA methylation. It was during the 1980s that a decrease in overall methylation levels was reported in the genomes of tumour cells [7]. This discovery was accompanied by the finding of oncogene activation through hypomethylation [8]. Paradoxically, a few years later hypermethylation and silencing of tumour suppressor genes were observed [9]. Another level of epigenetic regulation is the influence of non-coding genes on protein expression regulation. In relation to gene silencing, microRNAs and ncRNAs may act as effectors, decreasing mRNA and protein levels of their target genes, but they can also undergo epigenetic regulation by other mechanisms such as DNA promoter methylation.

This review will focus on how epigenetic alterations contribute to the silencing of tumour suppressor coding and non-coding genes with particular emphasis on recent discoveries. Molecular changes that are involved in carcinogenesis, such as aberrant division, immortality, genomic instability, metastasis, metabolic reprogramming and tumour plasticity will be analysed, highlighting genes silenced through epigenetic mechanisms.

## 2. Molecular epigenetics mechanisms

**Figure 1.**
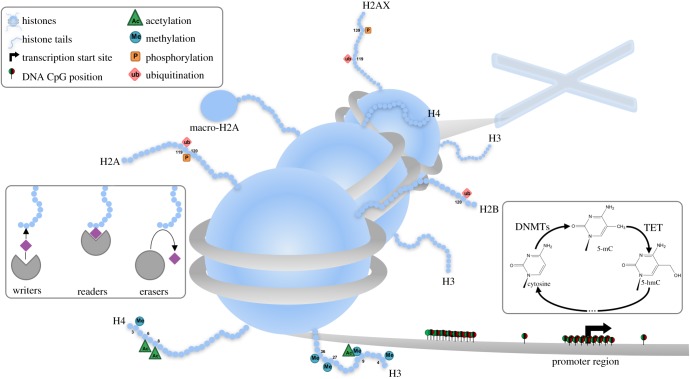
Covalent mechanisms of epigenetic regulation. DNA is bound to histone proteins forming the nucleosomes. Nucleosome compaction depends on histone tail modification, which is regulated by histone writers, readers and erasers. DNA methylation in CpG dinucleotides is regulated by different enzymes. DNMT1 and DNMT3a mediate the 5′mC synthesis. TET proteins catalyse DNA demethylation.

Various types of epigenetic mechanisms have been defined. The first group of these consist of covalent modifications of chromatin, affecting DNA and histones (figure 1). In mammals, DNA methylation occurs predominantly at the 5′ position of cytosine forming cytosine guanine dinucleotides (CpG). This modification is carried out by DNA methyltransferases (DNMTs), enzymes that use *S*-adenosylmethionine (SAM) as methyl group donor. In humans, DNMT1 is responsible for *de novo* methylation, whereas DNMT3a and DNMT3b are more related with methylation maintenance [10]. Demethylation can be carried out through 10–11-translocation proteins (TET). These enzymes can convert 5-methylcytosine (5mC) into 5-hydroxymethylcytosine (5hmC). The abundance of this hydroxylated nucleotide in promoter regions of genes seems to correlate with their active expression, while 5mC is generally related to transcription repression. 5hmC is finally converted back into cytosine by the action of TETs and other enzymes through the successive modification of the 5-residue, concluding the demethylation reaction [11]. DNA is not naked in the nucleus; it is associated with histones. Histones are grouped together to form octamers around which DNA is wrapped. Histones have an intrinsically unfolded domain, known as a histone tail, which can be highly modified. These modifications influence chromatin compaction, and may affect the binding affinity of different proteins and complexes for chromatin. According to histone marks, three groups of proteins are defined. Writers are proteins responsible for histone marks deposition while erasers act by removing these marks. Readers recognize these marks, and upon mark recognition they recruit various proteins with different functions that depend on chromatin context. The nature and position of the mark determines its role [12]. Thus, the same modification in one position may play an activating role, such as trimethylation in lysine 4 of histone 3 (H3K4Me3), but in another position may mediate a repressive action, such as trimethylation of lysine 9 of histone 3 (H3K9Me). On the other hand, modification of the same position with different tags may give rise to opposite effects: whereas tH3K9Me promotes repression, acetylation in the same position activates transcription. Beyond covalent modifications there are other epigenetic mechanisms to be considered. Among them there is the replacement of histones, such as macroH2A or H2AX [13,14]; ATP-dependent chromatin remodelling complexes, which are involved in nucleosome positioning; and non-coding RNAs (ncRNAS). ncRNAs participate in gene expression regulation in several ways. They can activate gene expression by forming DNA–RNA complexes, such as R-loops, but more often they interfere in mRNA translation by blocking ribosome binding or promoting mRNA degradation [15].

## 3. Aberrant division

The most prominent feature of tumour cells is their uncontrolled division. This ability comes from the deregulation of genes responsible for cell-cycle control, as well as genes related to signal transduction pathways involved in detecting external stimuli such as nutrients or mitogens. While alterations in cell-cycle genes often result in a lack of checkpoints, changes in transduction pathways eventually lead to pervasive activation of them in a stimulus-independent manner. The cell cycle is *broadly* controlled by cyclin-dependent kinases (CDKs). These enzymes catalyse phosphorylation reactions of different substrates in association with their regulatory subunits, the cyclin proteins, which increase CDK activity and contribute to target recognition. A well-programmed fluctuation of cyclin expression entails progression through different phases on the cell cycle. Contrary to what one might think, the critical step in cancer cells is neither the S-phase nor mitosis itself, but rather G_0_/G_1_ transition, when a cell decides its fate. During this phase, CDK4/6 control G_0_/G_1_ transition, and are activated by D cyclins (D1, D2, D3) [16]. Moreover, CDK activity is controlled by CDK inhibitors (CDKIs), which are also regulated by external stimuli. For instance, p15^INK4B^/CDKN2B inhibits CDK4/6 activity induced by TGF-β [17]. Thus, CDK4/6 inhibition, either by the lack of cyclins or by the activation of CDKIs, will result in a cessation of cellular proliferation. Cyclin expression is dependent on extracellular signal pathways that are often altered in cancer, such as Wnt/β-Catenin, RANK receptor, Shh/Patched or tyrosine kinase receptors. p16^INK4A^/CDKN2A and p15^INK4B^/CDKN2B, which are CDKIs for CDK4/6, are downregulated in several types of cancer [18]. In some, such as lymphoma, gastric cancer, head and neck squamous cell carcinoma (HNSC), liver and oesophageal cancers [18–22], p16^INK4A^/CDKN2A downregulation is caused by promoter hypermethylation, but it can also be due to histone deacetylase (HDAC) activity [23]. Retinoblastoma protein (pRB) is the master regulator of G_0_/G_1_ to S transition, translating cyclin fluctuations into transcription factor activation. pRB is hypophoshorylated during early G_1_ phase by CDK4/6 and hyperphosphorylated during late G_1_ by E-CDK2 complex. Low levels of phosphorylation allow pRB binding with E2F transcription factors, whereas pRB hyperphosphorylation liberates E2Fs from pRB control, enabling cell-cycle progression. pRB, which is encoded by *RB1* gene, is one of the most commonly mutated genes in cancer [24]. Apart from genetic mutations, epigenetic silencing has also been observed. Cells without functional pRB are not able to maintain themselves in G_0_/G_1_, and so undergo aberrant division [25,26]. *miR-124a*, *miR-129* and *miR-137* are downregulated through promoter hypermethylation on colorectal cancer (CRC) and breast cancer cell lines. These microRNAs target CDK6, so their silencing promotes CDK6 activation, which triggers increased phosphorylation of pRb by promoting cell-cycle progression [27–29].

Receptor tyrosine kinases (RTKs) provide one way by which cells sense their environment. Their ligand binding activation promotes conformational changes in their cytoplasmic domain releasing signals into the cytoplasm. MET is a RTK which is targeted by *miR-1-1* and *miR-34a* microRNAs. All of them are downregulated through promoter hypermethylation resulting in MET activation in some cancers [30,31]. IGF-1R is another RTK that is also regulated by *miR-214* [32] and *miR-345* [33], which in turn are aberrantly methylated in cancer [34]. *FGFR* expression is *negatively* controlled by *miR-9* family, but these microRNAs are frequently silenced by promoter hypermethylation in cancer [35]. Post-translational modifications of RTKs recruit a number of proteins that can activate RAS, promoting its GDP release and preferential GTP binding. RAS can activate the mitogen-activated protein kinase (MAPK) pathway by interacting with B-RAF, a MAPK pathway effector. B-RAF activates PERK, which in turn catalyses ERK phosphorylation. The MAPK pathway activates cytoplasmic and nuclear proteins involved in activating Jun and Fos transcription factors, as well as protein synthesis activation that contributes to cyclin D1 expression. Constitutive activation of the MAPK pathway is often observed in cancer cells due to epigenetic alterations of MAPK-related genes as well as mutations. For example, PTPRR, which dephosphorylates ERK, is methylated in cervical cancer [36]. DUSP1 and MKP1 are phosphatases which also target ERK, promoting MAPK pathway inactivation. The promoter regions of the *DUSP1* and *MKP1* genes have been found hypermethylated in oral squamous cell carcinoma and breast cancer, respectively [37,38]. *RAS* is directly regulated by *Let-7a* [39], a microRNA which is downregulated in different cancers such as head and neck cancer (HNC) through promoter hypermethylation [40].

The phosphatidylinositol 3-kinase (PI3K)/AKT pathway is also a RAS-dependent pathway. PI3K activation depends on its interaction with the GTP-bound form of RAS. Phosphatidylinositol (PI) is a component of the plasma membrane which can have a regulatory role that depends on its post-translational changes. After its phosphorylation by different kinases, such as PDK1, phosphatidylinositol-(4,5)-bisphosphate (PIP2) can be phosphorylated by PI3K, generating phosphatidylinositol-(3,4,5)-triphosphate (PIP3), which is recognized by AKT/PKB. PDK1 is targeted by *miR-375*, which is downregulated by promoter hypermethylation [41]. Active AKT/PKB contributes to cell proliferation by p21/CDKN1A, Tsc2 and GSK3-β inhibition [42]. The critical step for AKT/PKB activation is PIP3 formation. Alternatively, PIP2 can be cleaved by phospholipase C (PLC), generating diaciylglycerol (DAG) and inositol-(1,4,5)-triphosphate (IP3). PLCD1 has been found silenced in breast and gastric cancers by promoter hypermethylation [43]. It can be considered a tumour suppressor gene, as its activity reduces AKT/PKB activation and decreases PIP2 levels, which are related to metastatic events [43–45]. There is another family of proteins which interact with RAS, the Ras-association domain family (RASSF). These proteins interact with the activated form of RAS, often playing a different role in tumourigenesis, because they behave as tumour suppressors in contrast with PI3K or B-RAF. RASSF1 is the most famous member of this family. Its promoter has been found methylated in more than 30 tumour types [46,47]. RASSF1 activation promotes cyclin D1 downregulation by its interaction with p120E4F, which in turn interacts with p14^ARF^, pRB and p53 [48].

Apart from RTKs, the Wnt/β-Catenin signalling pathway is another canonical pathway altered through promoter hypermethylation in cancer cells. In the absence of Wnt signalling, β-Catenin is degraded by the GSK3-β, Axin and APC destruction complex. GSK3-β phosphorylates β-Catenin leading to its degradation by the proteasome. When Wnt interacts with Frizzled, Disheveled (DSH) is released and displaces GSK3-β, promoting β-Catenin activation. This protein is then translocated to the nucleus, where it binds Lef/TCF, promoting transcription of its target genes including *MYC* and *CNND1* (cyclin D1), both considered proto-oncogenes due to their capacity to enhance cell growth and cell proliferation [49]. Epigenetic alterations of this pathway generate high β-Catenin levels. Secreted frizzled related proteins (SFRP1, SFRP2, SFRP3), which are Frizzled competitors for Wnt, are silenced by promoter hypermethylation in different cancers, including hepatocellular carcinoma (HCC), lung adenocarcinoma, oesophageal squamous cell carcinoma (ESCC) and CRC [49–52]. NDRG2 and WIF-1, other Wnt regulators, are inactivated in pancreatic cancer and ESCC, respectively [53,54]. It is important to mention that some Wnt ligands cannot activate β-Catenin, and so act as Wnt/β-Catenin antagonists, examples of which include WNT5a and WNT7a, both of which are commonly silenced in CRC, pancreatic and lung cancer [55,56]. APC is frequently mutated in CRC, but it is also inactivated by promoter hypermethylation in colon, breast and pancreatic cancer [57–59]. NKD2, DACT2 and CXXC4 inhibit DSH. However, during tumourigenesis they are silenced by promoter hypermethylation [60,61]. ROR2 is a Wnt5a receptor of Frizzled independent pathway, which inhibits the canonical Wnt/β-Catenin pathway. This transmembrane protein is also downregulated in CRC though promoter hypermethylation [62].

Cancer cells often show myc activation through different mechanisms, such as myc amplification, but its activity can also be indirectly regulated through epigenetic events [63]. In acute myeloid leukaemia (AML), promoter hypermethylation of NUDT16, an RNA decapping enzyme, triggers an increase in c-myc half-life mRNA, contributing to its activation [64]. Extensive literature highlights the importance of epigenetic silencing events in tumour suppressor genes involved in cell-cycle control and mitogenic pathways.

## 4. Immortality

Tumour cells are considered immortal because they avoid cell death mechanisms that normally occur to avoid uncontrolled cell growth. This capacity can be given by telomerase activation through epigenetic deregulation or by the inhibition of pathways related to cell death. Tumour cells are subjected to different stress conditions, both extrinsic, such as oxygen and nutrient deprivation or death signals, and intrinsic, including DNA damage or ROS stress. These situations lead normal cells to die either by apoptosis or by other mechanisms. Therefore, tumour cells find ways to prevent cell death through inhibition of key genes involved in this programme. The crucial role of epigenetics in this reprogramming was observed by Kaminskyy *et al*. [65]. When they inhibited DNMTs and HDACs, apoptosis was reactivated. p53 is considered the master regulator of programmed cell death. Its activation, which is related to DNA damage or hypoxia, is highly regulated. One of these regulators is mouse double minute 2 (Mdm2), an E3-ubiquitin ligase which recognizes and mediates p53 degradation via the proteasome. Mdm2 transcription is upregulated by p53, generating a negative feedback, in order to avoid excessive p53 activity after its activation. Mdm2 activation depends on Akt/PKB phosphorylation, thereby connecting survival signals with the p53 regulation. Mdm2 is also regulated by p14/ARF, an E2F target gene that is often silenced in cancer through DNA hypermethylation of its promoter [66–68]. Thus, p53 deregulation can be explained in some cancers with p53 wild-type copies. When p53 is activated it promotes the transcription of growth arrest genes, such as p21/CDKN1A, a CDK inhibitor; 14-3-3 σ, which sequesters B-CDC2; and Reprimo, which promotes G2 arrest. 14-3-3 σ is silenced in a broad range of cancer types including nasopharyngeal carcinoma and breast cancer through promoter hypermethylation [69–71]. Meanwhile, Reprimo is also silenced by the same mechanism [72]. These epigenetic alterations allow cell-cycle progression in spite of p53 activation. TP53TG1 is a long-non-coding RNA which is regulated by p53. This RNA contributes to the DNA damage response (DDR) through its interaction with YBX1 preventing its nuclear localization. TP53TG1 is often downregulated through promoter hypermethylation, which triggers a poor outcome in gastrointestinal cancer patients [73].

If the stimulus that activates p53 ceases, for instance DNA damage is repaired or survival signals bind membrane receptors, p53 levels decrease, promoting cell-cycle progression. However, if the DNA damage cannot be repaired or if there is a sustained lack of nutrients, cells can enter into apoptosis. In this case p53 promotes the transcriptional activation of Bcl-2 superfamily genes. Within this heterogeneous family there are antiapoptotic proteins, such as Bcl-L1 and Bcl-2 itself as well as proapoptotic genes, including BAX and Only-bh3 family proteins [74]. Proapoptotic proteins participate in the formation of mitochondrial channels, contributing to cytochrome C release. Some proapoptotic proteins including BAX, BIM, Bid, HRK and PUMA are silenced in cancer [75,76]. microRNA-7, which targets the antiapoptotic Bcl-2, is also downregulated in lung cancer cells [77]. Whereas most of them are silenced through DNA promoter hypermethylation, Bid inhibition is driven by SIN3a/ HDAC1/2 corepressor complex [78]. Once cytochrome C is released it multimerizes with Apaf-1 and caspase 9, generating the apoptosome. Apaf-1 inhibition through DNA promoter hypermethylation allows apoptosis bypass in renal carcinomas [79]. Besides the intrinsic pathway, there is a p53-independent pathway, the extrinsic apoptosis pathway, that is activated by cytokines. When FAS receptor recognizes Fas ligand, or tumour necrosis factor-related apoptosis-inducing ligand (TRAIL) binds its receptor DR4 (death receptor), the apoptosis programme is initiated. Subsequently, the death-induced signalling complex (DISC) is formed by recruitment of FADD (Fas-interacting DD), proCasp8 and proCasp10 proteins. ProCasps are then activated and switch on the intrinsic pathway either through Bid activation by cleavage or through activating executioner caspases (Casp3,6,7). After its activation, Bid migrates to mitochondria where channels are opened and cytochrome C is released, becoming part of the apoptosome. During tumourigenesis death signals that can activate this pathway remain active, but the inactivation of the transduction proteins is a common feature among cancers. The DR4 receptor is inhibited by DNA methylation of its promoter region in different cancers [80,81]. Concerning Fas receptor, its regulation by DNA methylation is controversial. Butler *et al.* [82] described that Fas promoter hypermethylation did not explain Fas downregulation in CRC. Three years later, Petak *et al.* [83] demonstrated that methylation of the enhancer region leads to Fas downregulation in this type of cancer. In addition, Fas has been found methylated in bladder and oesophageal cancer [84,85]. Surprisingly, its promoter methylation status did not correlate with its expression in colorectal RKO cells, but its expression did correlate with the methylation status of the enhancer region. FADD and Casp8 were also downregulated by promoter hypermethylation [81,85].

TMS1/ASC is a bipartite protein that plays a role in apoptosis and in the NF-κB pathway. It may serve as an adaptor for Casp8 and Casp10 caspases or it can interfere with IKK complex formation, contributing to apoptosis by activating the caspase cascade as well as blocking antiapoptotic and proliferative gene expression. The TMS1/ASC promoter was described to be hypermethylated in several cancer subtypes [86,87]. HACE1 is a protein which mediates TNFR1 activation, promoting apoptosis or necroptosis activation. Necroptosis is another form of programmed cell death that triggers membrane rupture and inflammation without the need for caspase activation. HACE1 has been found methylated in HCC [88]. RIPK3 is also involved in this pathway, which in turn is also silenced through DNA promoter hypermethylation [89]. Epigenetic alterations related to the roles of p53 in mobilizing components of DNA repair machinery and inhibiting angiogenesis will be described in detail below in the Genomic instability and Tumour plasticity sections, respectively.

Overall, avoiding cell death is a key step for tumourigenesis, where epigenetic dysregulation plays an essential role, from p53 regulation to apoptosis and necroptosis effectors.

## 5. Genomic instability

In general, during cancer progression DNA methylation levels decrease. In some cancers, mutations in DNMTs and TET2 have been reported [90–92], but this is not a common feature. In most cases, the mechanisms that cause this phenomenon are unknown and do not seem to depend on any single pathway. Global DNA demethylation promotes genomic instability by various mechanisms. Eden *et al.* [93] demonstrated that mitotic recombination, which can lead to gene translocations and fusions, was increased in DNMT3a- and DNMT3b-deficient cells. In addition, DNA demethylation allows retrotransposon transcription, such as long interspaced nuclear elements (LINEs). LINE-1 transcription is activated after DNA demethylation, leading to the insertion of these elements into other genomic locations. During tumourigenesis, LINE-1 transcripts can cause gene disruption and can also function as alternative splicing sites, as novel promoters or as polyadenylation signals in a retrotransposition-independent manner [94]. LINE-1 internal bidirectional promoters can generate LINE-1 chimeric transcripts (LCTs) containing parts of genomic sequences surrounding the LINE-1 locus. Cruickshanks *et al.* [95] discovered that LCT13, a 300 kb LINE-1, was upregulated in cancer and contributed to TFPI-2 downregulation as an antisense gene, demonstrating an epigenetic inactivation caused by a LINE element.

DNA repair genes show a dual role during cancer progression. Its inactivation via mutations or epigenetic silencing promotes accumulation of repair errors, increasing tumour heterogeneity and provoking alterations which may confer different advantages to cancer cells [96]. Furthermore, a lack of DNA damage sensors or an efficient response to them can promote faster cell division and avoidance of apoptosis [97]. However, this damaged DNA becomes more fragile, so stimuli such as oxidative damage or certain drugs can be lethal for tumour cells, giving rise to its use as a therapeutic target [98].

The DDR begins with the recognition of damaged DNA by different sensors, which may recruit different mediators depending on the nature of the DNA damage detected. As well as activating DNA repair mechanisms, these mediators also participate in cell-cycle arrest, prioritizing DNA repair before replication or mitosis; if DNA damage cannot be removed, chronic DDR signalling may trigger cell death by apoptosis or cellular senescence. In humans, these DNA damage sensors are ataxia telangiectasia-mutated (ATM) and ataxia telangiectasia and Rad3-related protein (ATR), which interact with double strand break (DSB) lesions [99]. On the other hand, following DNA lesions that generate ssDNA, replicative protein A (RPA) interacts with the DNA, positioning ATR interacting protein (ATRIP) close to the site of damage. ATM has been found hypermethylated in breast cancer [100], even before the appearance of palpable lesions [66]. The two best-studied ATM/ATR targets are CHK1 and CHK2, which stop cell-cycle progression by reducing CDK activity through different mechanisms. CHK2 has been found hypermethylated in gliomas [101]. ATM/ATR also catalyses Ser139-γH2AX phosphorylation, located around DSB sites, promoting the recruitment of DDR factors.

DNA repair machinery is specific for each type of alteration. For example, direct DNA repair (DR) enzymes, base excision repair (BER), nuclear excision repair (NER) or mismatch repair (MMR) mechanisms are triggered when single nucleotide base damage occurs; by contrast, recombination repair mechanisms (non-homologous end joining (NHEJ) or homologous directed recombination (HDR)) are active when DSBs are induced. Deficiencies in DNA repair enzymes can be caused by somatic mutation, but are much more frequently caused by epigenetic alterations that reduce or silence their expression.

MGMT is an enzyme that directly repairs *O*-6-methylguanines. If it is inactive, unrepaired guanine will match with adenine instead of cytosine, giving rise to genomic instability. The MGMT promoter is often hypermethylated in oesophageal cancer and CRC [22,102], but its methylation status is especially taken into account in glioblastomas [103,104] because it is used as temozolomide response biomarker.

BER is a process which consists of the detection and correction of damaged bases in the DNA. This pathway is initiated by a number of glycosylases (e.g. Ogg1, MDB4, NEIL1), which differentially recognize and remove each aberrant base, generating an AP site (apurinic/apyrimidinic site). Ogg1 is a glycosylase that recognizes 8-oxoguanosine, being reported as inactivated by promoter DNA hypermethylation in breast cancer [105]. MBD4 recognizes thymidines or uracils produced by hydrolysis of cytosines in CpG sites, and it is methylated in CRC and ovarian cancer [106]. NEIL1 recognizes and removes oxidized pyrimidines. This gene is also methylated in certain types of cancer, such as HNC [107]. An AP endonuclease then cleaves the AP site, generating a single-strand break that can be processed by either a short-patch (just a nucleotide) or a long patch (from 5 to 10 nt). Finally, DNA replication machinery synthesis followed by XRCC1-ligase IIIα ligation ends the BER process.

NER is a similar process, which recognizes bulky DNA lesions produced by mutagens or UV irradiation. NER can be initiated by global genome NER (GG-NER) or transcription-coupled NER (TC-NER) on the transcribed strands of active genes [108]. In GG-NER, DNA lesions are mainly recognized by XPC-hRAD23B-CETN2. After recognition, this complex degrades the DNA surrounding the lesion and recruits TFIIH. By contrast, TC-NER is activated when RNA polymerase II (RNAPII) is blocked by bulky adducts. Different factors including CSA, CSB and XAB2 interact with blocked RNAPII, promoting its backtracking and recruitment of TFIIH, the convergent step between GG-NER and TC-NER [108]. XPC and RAD23B are epigenetically silenced in cancer through DNA promoter hypermethylation in lung and multiple myeloma, respectively [109,110]. After recognition, TFIIH complex proteins XPB and XPD unwind the DNA to create a 20- to 30-nt bubble. This event allows the recruitment of XPA, RPA, XPG and ERCC1–ERCC4/XPF. XPA binds the 5′ side of the bubble and RPA interacts with the complementary ssDNA that does not have the lesion. Next, ERCC1–ERCC4/XPF cuts the 5′ injured strand, and after patch synthesis by DNA replication machinery, XPG cuts the 3′ strand. Epigenetic silencing of ERCC1 has been described in HNC through DNA promoter hypermethylation [111]. Finally, XRCC1-ligase IIIα seals the DNA [108].

DNA MMR machinery is responsible for replacing mismatched Watson–Crick nucleotides. Mismatches are recognized by the MutS heterodimer, which can consist of either MSH2–MSH6 (MutSα) or MSH2–MSH3 (MutSβ). MutS is then stabilized by the MutL heterodimer, which in turn can be made of MLH1–PMS2 (MutLα), MLH1–PMS1 (MutLβ) or MLH1–MLH3 (MutLγ) [112]. The MutS–MutL complex recruits different proteins that can generate an incision closer to the DNA injury and carry out the removal of the damaged strand. The DNA replicative machinery then resynthesizes the damaged strand anew. Genes involved in MMR are often altered in hereditary non-polyposis CRC syndrome (Lynch syndrome). For instance, MLH1 inactivation by promoter hypermethylation is a common alteration in this pathology [113].

HDR normally is found inactivated in cancer cells. When DNA damage occurs, H2A is replaced by γH2AX in the nucleosomes near to the DNA lesion. After γH2AX phosphorylation by ATM, MDC1 is recruited. MDC1 promotes RNF168/RNF8 binding and MRN complex interaction, formed by MRE11-RAD50-NBS1. The MRN complex stimulates the kinase activity of ATM [114] and participates in 5′ → 3′ resection.

BRCA1 participates in HDR through RNF168/RNF8, a pair of E3-ubiquitin ligases that modify proteins at DSB sites. Next, BRCA1 mediates the 5′ → 3′ resection. After 5′ → 3′ resection by BRCA1, BRCA2 loads RAD-51 onto ssDNA, a necessary step for invading the homologous double helix. The homologous DNA is used as a template for damaged DNA strand resynthesis [115]. RAD51 is downregulated in renal cell carcinoma by histone methylation [116]. However, RAD51 seems to be overexpressed in many different types of cancer. RAD51 has five paralogues, which play essential roles in the HDR pathway [117]. Of these, RAD51B and XRCC3 are downregulated by DNA promoter hypermethylation in different cancer types, including HNSC, lung and cervix [118]. RNF168/RNF8 mediates BRCA1 or 53BP1 binding. BRCA1 is a key regulator of the HDR pathway and several mutations and epigenetic silencing events have been reported, especially in breast and ovarian cancers [119]. This inactivation leads to 53BP1 binding to DSB sites, activating NHEJ to the detriment of HDR [120]. BRCA1 can also be deregulated by epigenetic alterations of its partners, such as SRBC for which inactivation by promoter methylation has been described in CRC [121]. It has been also reported that some HDR-related helicases are silenced in different types of cancers, such as WRN, which is often methylated in cervical cancer [122]. DNA hypermethylation is also present on the SLFN11 promoter in different types of cancer [123]. SLFN11 is a putative helicase that interacts with DHX9, another BRCA1 interactor, and it has been postulated that SLFN11 destabilizes the DHX9 complex, thus impairing complete damage repair [124].

The alternative process to HDR following a DSB is NHEJ, which is more common during G_1_ phase. When a DNA lesion occurs, BRCA1 and 53BP1 compete for binding to the damage site. If 53BP1 interacts with phosphorylated γH2AX or with the MRN complex, BRCA1-mediated resection is blocked, and NHEJ starts [125]. The Ku70/Ku80 heterodimer can bind DSB ends (non-resected ends). After its binding, these proteins recruit XRCC4, which may serve as scaffold for DNA end processing enzymes, such as ligase IV, WRN or a DNA polymerase [126]. Ku70/Ku80 also recruits DNA-PKcs, which can also process the damaged region. Ku80 and DNA-PKc (also called XRCC5 and XRCC7, respectively) have been found hypermethylated in glioma [127].

These data prompt us to think that DNA repair system inhibition is a double-edged sword for cancer cells, increasing their division rate and mutation accumulation but sensitizing them to certain types of damage inflicted by UV, ionizing radiation and chemotherapeutic agents.

## 6. Metastasis

Cancer cells can develop several strategies to adapt themselves to new environments. Epithelial-to-mesenchymal transition (EMT) is one of the most dramatic changes, whereby epithelial cells obtain mesenchymal features. These cells are poorly differentiated, and they can detach from the primary tumour, migrate through the stromal environment and reach the bloodstream. The reversibility of this process allows transformed cells to undergo mesenchymal-to-epithelial transition, and then continue proliferating at distant localizations. Carmona *et al.* [128] demonstrated common DNA methylation switches in MDCK and MDA-MB-468 associated with EMT phenotype acquisition after TGF-β treatment, supporting the importance of epigenetic alterations during this process. TGF-β activates several pathways, promoting the expression of transcription factors (TFs) that direct cell reprogramming, including FOXOC1, TWIST and SNAIL [129]. Activation of these TFs positively correlates with the expression of vimentin, fibronectin and N-cadherin, all well-known mesenchymal markers. On the other hand, aberrant epigenetic silencing of TFs such as SOX1, KLF4, HIC1 and DACH1 through promoter hypermethylation has been reported in HCC, lung, gastric and urothelial cancer [130–133]. HOXA10, another epithelial-related TF, is silenced in breast cancer by CTCF insulator binding [134]. Several publications report that EMT reprogramming promotes epigenetic silencing of cell junction-related genes such as ITGA5, CDH11, CADM1 and OLFM4 [135–138]. Ectopic expression of these silenced genes decreased cell migration potential, highlighting their role in the EMT process. Post-translational modifications of membrane proteins may also contribute to cell adhesion. B3GNT7 is an *O*-glycosyltransferase that in CRC is downregulated by epigenetic silencing, and as in the cases listed above, its recovery decreases cells migration and invasion ability [139]. However, EMT is not a linear process and many different mechanisms are involved in its regulation. Thus, whereas ITGA2 is inhibited in some breast cancers by *miR-373* effect, *miR-373* silencing has been observed in lung cancer [140,141]. CDH1 is a cadherin protein involved in cell–cell adhesion. ZEB1 and ZEB2 are two transcriptional repressors of *CDH1*. These genes are regulated by *miR-200* microRNA family. Normal expression of *miR-200* may maintain CDH1 expression, but aberrant promoter hypermethylation of *miR-200* results in a CDH1 downregulation, which triggers cell migration [142]. In addition to cell detachment, another important step for cell invasion is extracellular matrix degradation, with activation of proteases being a common feature in invasive front cells [143]. Epigenetic silencing of protease inhibitor proteins has also been reported. For instance, SPINT2 is often silenced in gastric cancer, ESCC and melanoma by DNA promoter hypermethylation [144–146]. MMP-9 is a well-characterized metalloprotease, which is overexpressed in different cancers. Its higher activity may be partially explained by RECK or KISS1 silencing due to promoter hypermethylation. Whereas RECK is an extracellular protein with a metalloprotease inhibitor domain [147], KISS1 seems to be involved in interfering with NF-κB-mediated MMP-9 transcriptional activation [148]. *miR-145* targets MMP-11, and has been reported to be inactivated in different cancer malignancies. miR-145 ectopic expression suppressed cell invasion and migration in renal cell carcinoma [149]. mTOR, a molecular sensor of cellular status that is also involved in metastasis, integrates several signals including PI3K/AKT, Wnt/β-Catenin, Ras pathways and AMP/ATP ratio. Its activation is mainly produced by TSC1/TSC2 inhibition. mTOR can generate two different complexes, mTORC1 and mTORC2. mTORC1 is related to MMP-2 and MMP-9 activation, contributing to the invasiveness of cancer cells. mTORC1 is also related to anabolism, participating in protein synthesis and inhibiting autophagy. mTORC2 also seems to promote cell motility through activation of focal adhesion kinase (FAK) and Rho GTPases [150]. TSC1/TSC2, which partially contributes to mTOR regulation, is epigenetically silenced by metastasis associated 1 family, member 2 (MTA2) in association with EZH2, a component of polycomb repressor complex 2 [151]. PIP2 is a metabolite produced in the PI3K/AKT pathway, and plays an important role in cell motility. PIP2 participates in actin reorganization, becoming an interaction point for actin binding proteins. Actin reorganization is a necessary step for migration/invasion. PIP2 is hydrolysed by PLCD, a tumour suppressor gene methylated in cancer. MMP-7, another protease, also seems to be downregulated by PLCD, thereby decreasing its activity [43]. miR-345, which is downregulated in non-small cell lung cancer through promoter hypermethylation [152], targets IRF1, a downstream factor of mTOR/AKT signalling involved in the EMT process [153]. Taken together, these data demonstrate the importance of epigenetic marks during EMT in modifying not only transcription factors but also cell adhesion and invasion genes.

## 7. Metabolic reprogramming

Metabolic reprogramming is one of the most universal features of cell transformation. Cancer cells tend to enhance glucose uptake through HIF1a stabilization, promoting a GLUT1 increase. In some cancers, HIF1a stabilization may be promoted by VHL promoter hypermethylation [154]. Paradoxically, this increase in glucose uptake is not translated into a huge increase at ATP levels, but instead the additional glucose is mostly metabolized by glycolysis. This phenomenon, known as the Warburg effect, is defined as the anaerobic use of glucose regardless of the presence of oxygen, conferring enormous advantages to cancer cells. Glycolysis results in lactate production, causing acidification of the extracellular space and activation of proteases involved in cell migration/invasion [155]. Enzymatic shift by alternative splicing of PKM gene between isoforms PKM1 or PKM2 promotes metabolite accumulation, activating the pentose phosphate pathway (PPP) [156]. This pathway produces NADPH, an essential metabolite for glutathione and fatty acid synthesis, required for ROS protection and cell growth, respectively. The PPP also synthesizes ribonucleotides that are necessary for cell division [157]. Epigenetic silencing of certain genes may generate a glucose uptake increase. Lopez-Serra *et al.* [158] demonstrated that DERL3 targets GLUT1 in colorectal cells, leading to its proteasomal degradation. Ectopic DERL3 expression partially reverted the Warburg effect in HCT116 cells, decreasing the lactate production and increasing O_2_ consumption, as well as decreasing metastasis in mice [158]. Tricarboxylic acid cycle is an anaplerotic mitochondrial pathway mainly involved in obtaining energy from acetyl-CoA. α-Ketoglutarate, a metabolite of this pathway, is necessary for TET and histone demethylase (HDM) activity. Mutations of the IDH1 and IDH2 enzymes (isocitrate dehydrogenases) and epigenetic inactivation of glutaminase (GS) can decrease α-ketoglutarate levels, promoting aberrant methylation marks on the epigenome [159–162]. Glucose uptake also increases serine glycine one-carbon metabolism (SGOC) pathway activity, connected by 3-phosphoglycerate dehydrogenase (PHGDH). This complex network connects the folate and methionine cycles. Folate is necessary for amino acid and nucleotide synthesis. Inhibition of this pathway, which is upregulated in cancer, has been used as a therapeutic strategy for several years due to its role in cell division [163]. Moreover, the methionine cycle is involved in SAM synthesis. SAM is a necessary substrate for DNMTs and histone methyltransferases (HMTs). Thus, this metabolite encompasses epigenetic modifications that are often related to gene silencing and metabolism. In some cancers, SAM synthesis is decreased by epigenetic silencing of SGOC genes. MAT1 and MTHFR protein levels are decreased in early pre-neoplastic rat livers by *miR-22* and *miR-29b* alterations and histone modifications, promoting a decrease in SAM synthesis, suggesting a rate-limiting substrate for epigenome maintenance [164]. The Met cycle is also connected with cysteine transsulfuration pathway. Two enzymes of this pathway, cystathionine beta synthase (CBS) and cysteine dioxygenase type 1 (CDO1), are often silenced by epigenetic inactivation in gastric and breast cancer, respectively [165,166]. Transsulfuration pathway products glutathione and taurine are redox controllers. These epigenetic alterations may lead to a decrease in redox-controlling molecules, facilitating tumourigenesis. Besides glutathione synthesis deregulation, antioxidant enzymes such as superoxide dismutase 2 (SOD2) and glutathione peroxidase (GP3) are frequently inactivated by promoter hypermethylation in lung and renal cancer, respectively [167,168]. Reviewed data suggest that epigenetic inactivation contributes to metabolic reprogramming, tumour progression through mutation accumulation by ROS, and dysregulation of epigenetic marks by rate-limiting substrates such as SAM and α-ketoglutarate. All of these metabolic alterations, acquired through epigenetic alterations, confer a selective advantage on cancer cells.

## 8. Tumour plasticity

The Sonic Hedgehog (Shh) pathway is aberrantly activated in different cancer types. This pathway can affect both tumour cells and stromal cells [169]. Within the tumour, it is important to highlight its importance in cancer stem cells (CSCs) [170]. It has been demonstrated that the Shh pathway is necessary for renewal of CSCs [171]. In normal conditions, Shh ligand binds Patched (PTCD) transmembrane protein, allowing Smoothened (SMO) activation. HHIP is a Shh ligand antagonist that has been found silenced through promoter hypermethylation in HNSC [61]. PTCD can be epigenetically inactivated in cancer, promoting an Shh ligand-independent activity [172]. When SMO is activated, it inhibits GLI destruction complex, allowing its nucleus translocation. GLI activates the transcription of target genes involved in self renewal and EMT, such as SNAIL [173]. ZIC1 and ZIC4 are GLI antagonists, and they are also inhibited through promoter hypermethylation [61]. High-mobility-group proteins comprise a family of chromatin associated proteins that are involved in maintaining stem cell-like properties in CSCs. HMG2A is a member of this family, and has been associated with Wnt pathway activation [174] and EMT progression [175]. Overexpression of this gene in cancer cells in comparison with normal tissue can be partially explained because *Let-7a*, a microRNA that targets HMG2A, undergoes downregulation through promoter hypermethylation [176]. The Notch pathway is involved in cell–cell communication. Although its role in cancer is controversial due to its dual oncogenic and tumour suppressor properties, it has been demonstrated that epigenetics is involved in its regulation. Notch receptors recognize a number of ligands. This binding promotes migration of Notch intracellular domain into the nucleus and modification of gene expression. Four Notch receptors and five receptor ligands have been discovered. All receptors and four of the five ligands have been found methylated in different types of cancer [177].

## 9. Tumour suppressor genes epigenetic recovery in the fight against cancer

Cancer is not a single disease. There is no master alteration common to all cancer types. Epigenetic alterations may confer a selective advantage upon tumour cells, due to their potential reversibility. Many studies have been focused on cancer treatment by reversing these epigenetic events. DNA methylation inhibiting drugs were the first anti-epigenetic treatment, tested in leukaemias [178]. 5′-Aza-2′-deoxycytidine and decitabine are hypomethylating agents which can recover epigenetically silenced genes [179]. Alterations in histone modifications have also been therapeutic targets. Vorinostat was the first HDACi approved for clinical use, and several are currently undergoing clinical trials [180]. There also are preclinical trials with HATi, especially focused on Tip60 inhibition [181]. HMTs and HDMs have also been considered potential small molecule targets, including DOT1L, a HMT that has been inhibited by different drugs in MLL (mixed lineage leukaemia) [182], and LSD1, which is a HDM studied as a target in AML [182]. Besides targeting writers and erasers, inhibition of readers is also an area of ongoing investigation. Several bromodomain inhibitors have been developed, especially against the BET family [183].

The major limitation of these treatments is their lack of specificity. Blocking the enzymes responsible for depositing, reading or removing epigenetic marks entails global genomic effects with counterproductive side effects. For instance, a hypomethylating agent could reactivate a hypermethylated tumour suppressor gene but may also promote the expression of an oncogene at the same time. In the near future, this limitation may be overcome by CRISPR-mediated epigenetic editing, allowing site-specific epigenetic modification. Indeed there are some interesting results in basic research, although they are still far from the clinic [184]. Despite the lack of widespread use of epigenetic drugs in cancer therapy, epigenetics has other clinical applications. DNA methylation, due its stability in comparison with mRNA, is used as a biomarker for diagnosis [185], cancer monitoring [186], cancer prediction [187], cancer prognosis [188] and treatment response [189].

## 10. Concluding remarks

**Figure 2.**
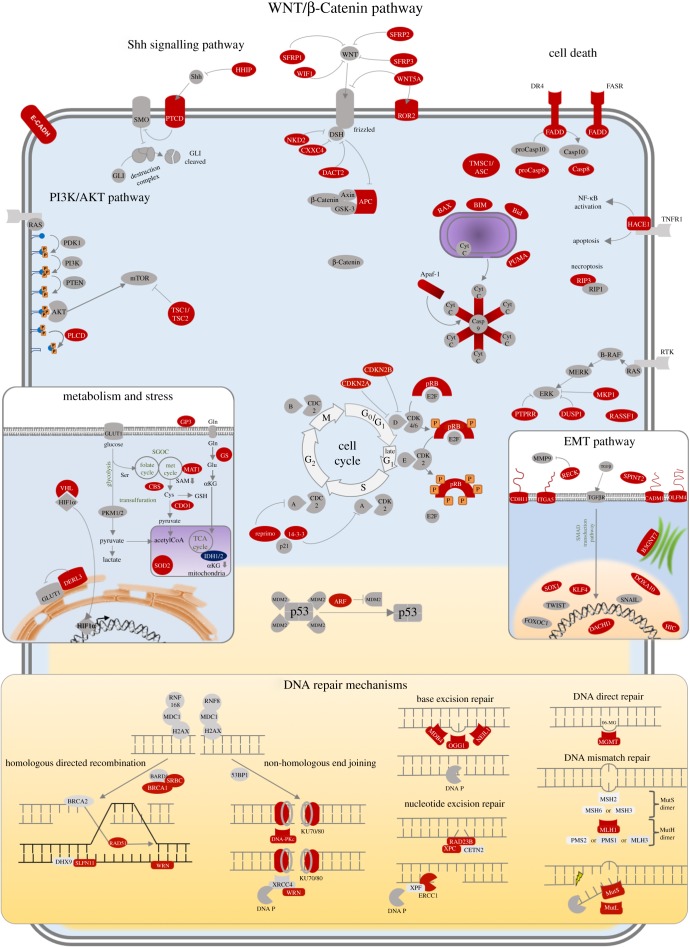
Molecular pathways altered by epigenetic inactivation in cancer. Represented pathways are altered in cancer. Red proteins correspond to epigenetically silenced genes.

‘To defeat your enemy, you must know your enemy’ [190]. This proverb, which derives from *The Art of War* by Sun-tzu, is applicable to one of the most important challenges of this century: to understand cancer at the molecular level. Since epigenetics was first described, many studies have contributed to decipher its implications in tumour transformation, particularly with regard to how the genes that are responsible for keeping cells under control are silenced. Tumour cells progressively acquire perturbations that allow them to divide without control, to adapt themselves to unfavourable conditions and even to abandon their niche and colonize other tissues (summarized in figure 2).

In this review, we have provided a global update on this knowledge, highlighting those coding and non-coding tumour suppressor genes whose epigenetic inactivation gives rise to proliferative advantages (listed in table 1), by rewiring the most important pathways by which cancer cells perpetuates themselves.

**Table 1.** Coding and non-coding genes silenced in cancer.

gene	epigenetically inactivated	involved in	reference
14-3-3 σ	promoter hypermethylation	immortality	[70,71]
Apaf-1	promoter hypermethylation	immortality	[79]
APC	promoter hypermethylation	aberrant division	[57]
ATM	promoter hypermethylation	genomic instability	[100]
BAX	promoter hypermethylation	immortality	[75]
BIM	HDAC activity	immortality	[78]
BRCA1	promoter hypermethylation	genomic instability	[119]
CADM1	promoter hypermethylation	metastasis	[137]
Casp 8	promoter hypermethylation	immortality	[81]
CBS	promoter hypermethylation	metabolism and stress	[165]
CDH11	promoter hypermethylation	metastasis	[136]
CDO1	promoter hypermethylation	metabolism and stress	[166]
CHK2	promoter hypermethylation	genomic instability	[101]
CXXC4	promoter hypermethylation	aberrant division	[61]
DACH1	promoter hypermethylation	metastasis	[133]
DACT2	promoter hypermethylation	aberrant division	[61]
DERL3	promoter hypermethylation	metabolism and stress	[158]
DNA-PKc	promoter hypermethylation	genomic instability	[127]
DR4	promoter hypermethylation	immortality	[80]
DUSP1	promoter hypermethylation	aberrant division	[37]
ERCC1	promoter hypermethylation	genomic instability	[111]
FADD	promoter hypermethylation	immortality	[85]
FASR	promoter hypermethylation	immortality	[83]
GP3	promoter hypermethylation	metabolism and stress	[168]
GS	promoter hypermethylation	metabolism and stress	[161]
HACE1	promoter hypermethylation	immortality	[88]
HHIP	promoter hypermethylation	tumour plasticity	[61]
HIC1	promoter hypermethylation	metastasis	[132]
HOXA10	CTCF binding	metastasis	[134]
ITGA2	miR-373	metastasis	[140]
ITGA5	promoter hypermethylation	metastasis	[135]
KISS1	promoter hypermethylation	metastasis	[148]
KLF4	promoter hypermethylation	metastasis	[131]
Ku80	promoter hypermethylation	genomic instability	[127]
Let-7a	promoter hypermethylation	aberrant division, tumour plasticity	[39,176]
MAT1	histone modification, miR	metabolism and stress	[164]
MBD4	promoter hypermethylation	genomic instability	[106]
MGMT	promoter hypermethylation	genomic instability	[22]
miR-124a	promoter hypermethylation	aberrant division	[27]
miR-129	promoter hypermethylation	aberrant division	[28]
miR-137	promoter hypermethylation	aberrant division	[29]
miR-145	promoter hypermethylation	metastasis	[149]
miR-200	promoter hypermethylation	metastasis	[142]
miR-214	promoter hypermethylation	aberrant division	[32]
miR-345	promoter hypermethylation	aberrant division	[33]
miR-34a	promoter hypermethylation	aberrant division	[31]
miR-373	promoter hypermethylation	metastasis	[141]
miR-375	promoter hypermethylation	aberrant division	[41]
miR-7	promoter hypermethylation	immortality	[77]
miR-9	promoter hypermethylation	aberrant division	[35]
miR1-1	promoter hypermethylation	aberrant division	[30]
MLH1	promoter hypermethylation	genomic instability	[113]
MTHFR	histone modification, miR	metabolism and stress	[164]
NDRG2	promoter hypermethylation	aberrant division	[53]
NEIL1	promoter hypermethylation	genomic instability	[107]
NKD2	promoter hypermethylation	aberrant division	[60]
Notch L	promoter hypermethylation	tumour plasticity	[177]
NotchR	promoter hypermethylation	tumour plasticity	[177]
NUDT16	promoter hypermethylation	aberrant division	[64]
OGG1	promoter hypermethylation	genomic instability	[105]
OLFM4	promoter hypermethylation	metastasis	[138]
P14/ARF	promoter hypermethylation	immortality	[66]
P15INK4b/CDKN2B	promoter hypermethylation	aberrant division	[18]
P16INK4a/CDKN2A	promoter hypermethylation, HDAC activity	aberrant division	[18,23]
PLCD1	promoter hypermethylation	aberrant division	[43]
PTCD	promoter hypermethylation	tumour plasticity	[172]
PTPRR	promoter hypermethylation	aberrant division	[36]
RAD23B	promoter hypemethylation	genomic instability	[110]
RAD51	histone methylation	genomic instability	[116]
RAD51B	promoter hypermethylation	genomic instability	[118]
RASSF1	promoter hypermethylation	aberrant division	[46]
RB1	promoter hypermethylation	aberrant division	[24]
RECK	promoter hypermethylation	metastasis	[147]
Reprimo	promoter hypermethylation	immortality	[72]
RIPK3	promoter hypermethylation	immortality	[89]
ROR2	promoter hypermethylation	aberrant division	[62]
SFRP1	promoter hypermethylation	aberrant division	[50]
SFRP2	promoter hypermethylation	aberrant division	[51]
SFRP3	promoter hypermethylation	aberrant division	[52]
SLFN11	promoter hypermethylation	genomic instability	[123]
SOD2	promoter hypermethylation	metabolism and stress	[167]
SOX1	promoter hypermethylation	metastasis	[130]
SPINT2	promoter hypermethylation	metastasis	[146]
SRBC	promoter hypermethylation	genomic instability	[121]
TFPI-2	LCT activation	genomic instability	[95]
TMS1/ASC	promoter hypermethylation	immortality	[86,87]
TP53TG1	promoter hypermethylation	immortality	[73]
TSC1/TSC2	MTA2/EZH2	metastasis	[151]
VHL	promoter hypermethylation	metabolism and stress	[154]
WIF1	promoter hypermethylation	aberrant division	[54]
WNT5A	promoter hypermethylation	aberrant division	[55]
WNT7A	promoter hypermethylation	aberrant division	[56]
WRN	promoter hypermethylation	genomic instability	[122]
XPC	promoter hypermethylation	genomic instability	[109]
XRCC3	promoter hypermethylation	genomic instability	[118]
ZIC1	promoter hypermethylation	tumour plasticity	[61]
ZIC4	promoter hypermethylation	tumour plasticity	[61]

## Data accessibility

This article has no additional data.

## References

[1] Mukherjee S 2011 The emperor of all maladies. New York, NY: Simon and Schuster.

[2] Huebner RJ, Todaro GJ 1969 Oncogenes of RNA tumor viruses as determinants of cancer. Proc. Natl Acad. Sci. USA 64, 1087–1094. (doi:10.1073/pnas.64.3.1087) 5264139 10.1073/pnas.64.3.1087 PMC223347

[3] Aaronson S, Todaro S 1969 Human diploid cell transformation by DNA extracted from the tumor virus SV40. Science 166, 390–391. (doi:10.1126/science.166.3903.390) 4309480 10.1126/science.166.3903.390

[4] Knudson AG 1971 Mutation and cancer: statistical study of retinoblastoma. Proc. Natl Acad. Sci. USA 68, 820–823. (doi:10.1073/pnas.68.4.820) 5279523 10.1073/pnas.68.4.820 PMC389051

[5] Waddington CH 1939 Preliminary notes on the development of the wings in normal and mutant strains of *Drosophila*. Proc. Natl Acad. Sci. USA 25, 299–307. (doi:10.1073/pnas.25.7.299) 16577903 10.1073/pnas.25.7.299 PMC1077909

[6] Esteller M 2008 Epigenetics in cancer. N. Engl. J. Med. 358, 1148–1159. (doi:10.1056/NEJMra072067) 18337604 10.1056/NEJMra072067

[7] Feinberg AP, Vogelstein B 1983 Hypomethylation distinguishes genes of some human cancers from their normal counterparts. Nature 301, 89–92. (doi:10.1038/301089a0) 6185846 10.1038/301089a0

[8] Feinberg AP, Vogelstein B 1983 Hypomethylation of ras oncogenes in primary human cancers. Biochem. Biophys. Res. Commun. 111, 47–54. (doi:10.1016/S0006-291X(83)80115-6) 6187346 10.1016/s0006-291x(83)80115-6

[9] Greger V, Passarge E, Höpping W, Messmer E, Horsthemke B 1989 Epigenetic changes may contribute to the formation and spontaneous regression of retinoblastoma. Hum. Genet. 83, 155–158. (doi:10.1007/BF00286709) 2550354 10.1007/BF00286709

[10] Poh WJ, Wee CPP, Gao Z 2016 DNA methyltransferase activity assays: advances and challenges. Theranostics 6, 369–391. (doi:10.7150/thno.13438) 26909112 10.7150/thno.13438 PMC4737724

[11] Huang Y, Rao A 2014 Connections between TET proteins and aberrant DNA modification in cancer. Trends Genet. 30, 464–474. (doi:10.1016/j.tig.2014.07.005) 25132561 10.1016/j.tig.2014.07.005 PMC4337960

[12] Simó-Riudalbas L, Esteller M 2015 Targeting the histone orthography of cancer: drugs for writers, erasers and readers. Br. J. Pharmacol. 172, 2716–2732. (doi:10.1111/bph.12844) 25039449 10.1111/bph.12844 PMC4439870

[13] Cantariño N, Douet J, Buschbeck M 2013 MacroH2A—an epigenetic regulator of cancer. Cancer Lett. 336, 247–252. (doi:10.1016/j.canlet.2013.03.022) 23531411 10.1016/j.canlet.2013.03.022

[14] Gerić M., Gajski G, Garaj-Vrhovac V 2014 γ-H2AX as a biomarker for DNA double-strand breaks in ecotoxicology. Ecotoxicol. Environ. Saf. 105, 13–21. (doi:10.1016/j.ecoenv.2014.03.035) 24780228 10.1016/j.ecoenv.2014.03.035

[15] Esteller M 2011 Non-coding RNAs in human disease. Nat. Rev. Genet. 12, 861–874. (doi:10.1038/nrg3074) 22094949 10.1038/nrg3074

[16] Fisher RP 2016 Getting to S: CDK functions and targets on the path to cell-cycle commitment. F1000Res 5, 2374 (doi:10.12688/f1000research.9463.1) 27746911 10.12688/f1000research.9463.1 PMC5040153

[17] Liu F, Korc M 2012 Cdk4/6 inhibition induces epithelial–mesenchymal transition and enhances invasiveness in pancreatic cancer cells. Mol. Cancer Ther. 11, 2138–2148. (doi:10.1158/1535-7163.MCT-12-0562) 22869556 10.1158/1535-7163.MCT-12-0562 PMC3752412

[18] Krajnović M, Radojković M, Davidović R, Dimitrijević B, Krtolica K 2013 Prognostic significance of epigenetic inactivation of p16, p15, MGMT and DAPK genes in follicular lymphoma. Med. Oncol. 30, 441 (doi:10.1007/s12032-012-0441-3) 23275143 10.1007/s12032-012-0441-3

[19] Peng D, Zhang H, Sun G 2014 The relationship between P16 gene promoter methylation and gastric cancer: a meta-analysis based on Chinese patients. J. Cancer Res. Ther. 10, 292–295. (doi:10.4103/0973-1482.151535) 25693938 10.4103/0973-1482.151535

[20] El-Naggar AK, Lai S, Clayman G, Lee JK, Luna MA, Goepfert H, Batsakis JG 1997 Methylation, a major mechanism of p16/CDKN2 gene inactivation in head and neck squamous carcinoma. Am. J. Pathol. 151, 1767–1774. 9403727 PMC1858347

[21] Hinrichsen I, Kemp M, Peveling-Oberhag J, Passmann S, Plotz G, Zeuzem S, Brieger A 2014 Promoter methylation of MLH1, PMS2, MSH2 and p16 is a phenomenon of advanced-stage HCCs. PLoS ONE 9, e84453 (doi:10.1371/journal.pone.0084453) 24400091 10.1371/journal.pone.0084453 PMC3882222

[22] Wang J, Sasco AJ, Fu C, Xue H, Guo G, Hua Z, Zhou Q, Jiang Q, Xu B 2008 Aberrant DNA methylation of P16, MGMT, and hMLH1 genes in combination with MTHFR C677T genetic polymorphism in esophageal squamous cell carcinoma. Cancer Epidemiol. Biomarkers Prev. 17, 118–125. (doi:10.1158/1055-9965.EPI-07-0733) 18199718 10.1158/1055-9965.EPI-07-0733

[23] Kim JK et al. 2013 Targeted inactivation of HDAC2 restores p16INK4a activity and exerts antitumor effects on human gastric cancer. Mol. Cancer Res. 11, 62–73. (doi:10.1158/1541-7786.MCR-12-0332) 23175521 10.1158/1541-7786.MCR-12-0332

[24] Chinnam M, Goodrich DW 2011 RB1, development, and cancer. Curr. Top. Dev. Biol. 94, 129–169. (doi:10.1016/B978-0-12-380916-2.00005-X) 21295686 10.1016/B978-0-12-380916-2.00005-X PMC3691055

[25] Quiñonez-Silva G, Dávalos-Salas M, Recillas-Targa F, Ostrosky-Wegman P, Aranda DA, Benítez-Bribiesca L 2016 Monoallelic germline methylation and sequence variant in the promoter of the RB1 gene: a possible constitutive epimutation in hereditary retinoblastoma. Clin. Epigenetics 8, 1 (doi:10.1186/s13148-015-0167-0) 26753011 10.1186/s13148-015-0167-0 PMC4706693

[26] McCormick TM, Canedo NHS, Furtado YL, Silveira FA, de Lima RJ, Rosman ADF, Almeida Filho GL, Carvalho MG 2015 Association between human papillomavirus and Epstein-Barr virus DNA and gene promoter methylation of RB1 and CDH1 in the cervical lesions: a transversal study. Diagn. Pathol. 10, 59 (doi:10.1186/s13000-015-0283-3) 26032781 10.1186/s13000-015-0283-3 PMC4450846

[27] Lujambio A et al. 2007 Genetic unmasking of an epigenetically silenced microRNA in human cancer cells. Cancer Res. 67, 1424–1429. (doi:10.1158/0008-5472.CAN-06-4218) 17308079 10.1158/0008-5472.CAN-06-4218

[28] Yu X, Song H, Xia T, Han S, Xiao B, Luo L, Xi Y, Guo J 2013 Growth inhibitory effects of three miR-129 family members on gastric cancer. Gene 532, 87–93. (doi:10.1016/j.gene.2013.09.048) 24055727 10.1016/j.gene.2013.09.048

[29] Zhu X, Li Y, Shen H, Li H, Long L, Hui L, Xu W 2013 miR-137 inhibits the proliferation of lung cancer cells by targeting Cdc42 and Cdk6. FEBS Lett. 587, 73–81. (doi:10.1016/j.febslet.2012.11.004) 23178712 10.1016/j.febslet.2012.11.004

[30] Reid JF et al. 2012 miRNA profiling in colorectal cancer highlights miR-1 involvement in MET-dependent proliferation. Mol. Cancer Res. 10, 504–515. (doi:10.1158/1541-7786.MCR-11-0342) 22343615 10.1158/1541-7786.MCR-11-0342

[31] Li N, Fu H, Tie Y, Hu Z, Kong W, Wu Y, Zheng X 2009 miR-34a inhibits migration and invasion by down-regulation of c-Met expression in human hepatocellular carcinoma cells. Cancer Lett. 275, 44–53. (doi:10.1016/j.canlet.2008.09.035) 19006648 10.1016/j.canlet.2008.09.035

[32] Das F, Dey N, Bera A, Kasinath BS, Ghosh-Choudhury N, Choudhury GG 2016 MicroRNA-214 reduces Insulin-like Growth Factor-1 (IGF-1) receptor expression and downstream mTORC1 signaling in renal carcinoma cells. J. Biol. Chem. 291, 14 662–14 676. (doi:10.1074/jbc.M115.694331) 10.1074/jbc.M115.694331 PMC4938186 27226530

[33] Kong KL, Kwong DLW, Chan TH-M, Law SY-K, Chen L, Li Y, Qin Y-R, Guan X-Y 2012 MicroRNA-375 inhibits tumour growth and metastasis in oesophageal squamous cell carcinoma through repressing insulin-like growth factor 1 receptor. Gut 61, 33–42. (doi:10.1136/gutjnl-2011-300178) 21813472 10.1136/gutjnl-2011-300178

[34] Misiewicz-Krzeminska I et al. 2013 Restoration of microRNA-214 expression reduces growth of myeloma cells through positive regulation of P53 and inhibition of DNA replication. Haematologica 98, 640–648. (doi:10.3324/haematol.2012.070011) 23100276 10.3324/haematol.2012.070011 PMC3659997

[35] Rodriguez-Otero P et al. 2011 Deregulation of FGFR1 and CDK6 oncogenic pathways in acute lymphoblastic leukaemia harbouring epigenetic modifications of the MIR9 family. Br. J. Haematol. 155, 73–83. (doi:10.1111/j.1365-2141.2011.08812.x) 21810092 10.1111/j.1365-2141.2011.08812.x

[36] Su P-H et al. 2013 Epigenetic silencing of PTPRR activates MAPK signaling, promotes metastasis and serves as a biomarker of invasive cervical cancer. Oncogene 32, 15–26. (doi:10.1038/onc.2012.29) 22330137 10.1038/onc.2012.29

[37] Khor GH, Froemming GRA, Zain RB, Abraham MT, Omar E, Tan SK, Tan AC, Vincent-Chong VK, Thong KL 2013 DNA methylation profiling revealed promoter hypermethylation-induced silencing of p16, DDAH2 and DUSP1 in primary oral squamous cell carcinoma. Int. J. Med. Sci. 10, 1727–1739. (doi:10.7150/ijms.6884) 24155659 10.7150/ijms.6884 PMC3805925

[38] Chen F-M, Chang H-W, Yang S-F, Huang Y-F, Nien P-Y, Yeh Y-T, Hou M-F 2012 The mitogen-activated protein kinase phosphatase-1 (MKP-1) gene is a potential methylation biomarker for malignancy of breast cancer. Exp. Mol. Med. 44, 356–362. (doi:10.3858/emm.2012.44.5.040) 22333693 10.3858/emm.2012.44.5.040 PMC3366329

[39] Johnson SM et al. 2005 RAS is regulated by the let-7 microRNA family. Cell 120, 635–647. (doi:10.1016/j.cell.2005.01.014) 15766527 10.1016/j.cell.2005.01.014

[40] Yu C-C et al. 2011 MicroRNA let-7a represses chemoresistance and tumourigenicity in head and neck cancer via stem-like properties ablation. Oral Oncol. 47, 202–210. (doi:10.1016/j.oraloncology.2010.12.001) 21292542 10.1016/j.oraloncology.2010.12.001

[41] Li X, Lin R, Li J 2011 Epigenetic silencing of microRNA-375 regulates PDK1 expression in esophageal cancer. Dig. Dis. Sci. 56, 2849–2856. (doi:10.1007/s10620-011-1711-1) 21533613 10.1007/s10620-011-1711-1

[42] Carnero A 2010 The PKB/AKT pathway in cancer. Curr. Pharm. Des. 16, 34–44. (doi:10.2174/138161210789941865) 20214616 10.2174/138161210789941865

[43] Song J-J, Liu Q, Li Y, Yang Z-S, Yang L, Xiang T-X, Ren G-S, Chen J-B 2012 Epigenetic inactivation of PLCD1 in chronic myeloid leukemia. Int. J. Mol. Med. 30, 179–184. (doi:10.3892/ijmm.2012.970) 22576628 10.3892/ijmm.2012.970

[44] Hu X-T et al. 2009 Phospholipase C delta 1 is a novel 3p22.3 tumor suppressor involved in cytoskeleton organization, with its epigenetic silencing correlated with high-stage gastric cancer. Oncogene 28, 2466–2475. (doi:10.1038/onc.2009.92) 19448674 10.1038/onc.2009.92

[45] Xiang T, Li L, Fan Y, Jiang Y, Ying Y, Putti TC, Tao Q, Ren G 2010 PLCD1 is a functional tumor suppressor inducing G(2)/M arrest and frequently methylated in breast cancer. Cancer Biol. Ther. 10, 520–527. (doi:10.4161/cbt.10.5.12726) 20657189 10.4161/cbt.10.5.12726

[46] Hesson LB, Cooper WN, Latif F 2007 The role of RASSF1A methylation in cancer. Dis. Markers 23, 73–87. (doi:10.1155/2007/291538) 17325427 10.1155/2007/291538 PMC3850810

[47] Dammann R, Schagdarsurengin U, Seidel C, Strunnikova M, Rastetter M, Baier K, Pfeifer GP 2005 The tumor suppressor RASSF1A in human carcinogenesis: an update. Histol. Histopathol. 20, 645–663. (doi:10.14670/HH-20.645) 15736067 10.14670/HH-20.645

[48] Fenton SL et al. 2004 Identification of the E1A-regulated transcription factor p120 E4F as an interacting partner of the RASSF1A candidate tumor suppressor gene. Cancer Res. 64, 102–107. (doi:10.1158/0008-5472.CAN-03-2622) 14729613 10.1158/0008-5472.can-03-2622

[49] Baron R, Kneissel M 2013 WNT signaling in bone homeostasis and disease: from human mutations to treatments. Nat. Med. 19, 179–192. (doi:10.1038/nm.3074) 23389618 10.1038/nm.3074

[50] Meng Y, Wang Q-G, Wang J-X, Zhu S-T, Jiao Y, Li P, Zhang S-T 2011 Epigenetic inactivation of the SFRP1 gene in esophageal squamous cell carcinoma. Dig. Dis. Sci. 56, 3195–3203. (doi:10.1007/s10620-011-1734-7) 21567192 10.1007/s10620-011-1734-7

[51] Hao X-W, Zhu S-T, He Y-L, Li P, Wang Y-J, Zhang S-T 2012 Epigenetic inactivation of secreted frizzled-related protein 2 in esophageal squamous cell carcinoma. World J. Gastroenterol. 18, 532–540. (doi:10.3748/wjg.v18.i6.532) 22363119 10.3748/wjg.v18.i6.532 PMC3280398

[52] Schlensog M et al. In press. Epigenetic loss of putative tumor suppressor SFRP3 correlates with poor prognosis of lung adenocarcinoma patients. Epigenetics. (doi:10.1080/15592294.2016.1229730) 10.1080/15592294.2016.1229730 PMC5997146 27623992

[53] Yamamura A et al. 2017 NDRG2, suppressed expression associates with poor prognosis in pancreatic cancer, is hypermethylated in the second promoter in human gastrointestinal cancers. Biochem. Biophys. Res. Commun. 484, 138–143. (doi:10.1016/j.bbrc.2017.01.055) 28093228 10.1016/j.bbrc.2017.01.055

[54] Yang S-H, Li S-L, Dong Z-M, Kan Q-C 2012 Epigenetic inactivation of Wnt inhibitory factor-1 in human esophageal squamous cell carcinoma. Oncol. Res. 20, 123–130. (doi:10.3727/096504012X13477145153039) 23193918 10.3727/096504012x13477145153039

[55] Li J, Ying J, Fan Y, Wu L, Ying Y, Chan AT. C., Srivastava G, Tao Q 2010 WNT5A antagonizes WNT/β-catenin signaling and is frequently silenced by promoter CpG methylation in esophageal squamous cell carcinoma. Cancer Biol. Ther. 10, 617–624. (doi:10.4161/cbt.10.6.12609) 20603606 10.4161/cbt.10.6.12609

[56] Kim T-H, Moon J-Y, Kim S-H, Paik SS, Yoon HJ, Shin DH, Park SS, Sohn JW 2015 Clinical significance of aberrant Wnt7a promoter methylation in human non-small cell lung cancer in Koreans. J. Korean Med. Sci. 30, 155–161. (doi:10.3346/jkms.2015.30.2.155) 25653486 10.3346/jkms.2015.30.2.155 PMC4310941

[57] Michailidi C, Theocharis S, Tsourouflis G, Pletsa V, Kouraklis G, Patsouris E, Papavassiliou AG, Troungos C 2015 Expression and promoter methylation status of hMLH1, MGMT, APC, and CDH1 genes in patients with colon adenocarcinoma. Exp. Biol. Med. 240, 1599–1605. (doi:10.1177/1535370215583800) 10.1177/1535370215583800 PMC4935349 25908636

[58] Swellam M, Abdelmaksoud MDE, Sayed Mahmoud M, Ramadan A, Abdel-Moneem W, Hefny MM 2015 Aberrant methylation of APC and RARβ2 genes in breast cancer patients. IUBMB Life 67, 61–68. (doi:10.1002/iub.1346) 25684670 10.1002/iub.1346

[59] Ginesta MM et al. 2016 APC promoter is frequently methylated in pancreatic juice of patients with pancreatic carcinomas or periampullary tumors. Oncol. Lett. 12, 2210–2216. (doi:10.3892/ol.2016.4868) 27602165 10.3892/ol.2016.4868 PMC4998563

[60] Cao B, Yang W, Jin Y, Zhang M, He T, Zhan Q, Herman JG, Zhong G, Guo M 2016 Silencing NKD2 by promoter region hypermethylation promotes esophageal cancer progression by activating Wnt signaling. J. Thorac. Oncol. 11, 1912–1926. (doi:10.1016/j.jtho.2016.06.015) 27374455 10.1016/j.jtho.2016.06.015

[61] Paluszczak J, Wiśniewska D, Kostrzewska-Poczekaj M, Kiwerska K, Grénman R, Mielcarek-Kuchta D, Jarmuż-Szymczak M 2017 Prognostic significance of the methylation of Wnt pathway antagonists-CXXC4, DACT2, and the inhibitors of sonic hedgehog signaling-ZIC1, ZIC4, and HHIP in head and neck squamous cell carcinomas. Clin. Oral Investig. 21, 1777 (doi:10.1007/s00784-016-1946-5) 10.1007/s00784-016-1946-5 PMC5442212 27553089

[62] Ma SSQ, Srivastava S, Llamosas E, Hawkins NJ, Hesson LB, Ward RL, Ford CE 2016 ROR2 is epigenetically inactivated in the early stages of colorectal neoplasia and is associated with proliferation and migration. BMC Cancer 16, 508 (doi:10.1186/s12885-016-2576-7) 27440078 10.1186/s12885-016-2576-7 PMC4955198

[63] Kalkat M, De Melo J, Hickman KA, Lourenco C, Redel C, Resetca D, Tamachi A, Tu WB, Penn LZ 2017 MYC deregulation in primary human cancers. Genes 8, 151 (doi:10.3390/genes8060151) 10.3390/genes8060151 PMC5485515 28587062

[64] Anadón C et al. 2017 Epigenetic loss of the RNA decapping enzyme NUDT16 mediates C-MYC activation in T-cell acute lymphoblastic leukemia. Leukemia 31, 1622–1625. (doi:10.1038/leu.2017.99) 28344317 10.1038/leu.2017.99 PMC5501321

[65] Kaminskyy VO, Surova OV, Vaculova A, Zhivotovsky B 2011 Combined inhibition of DNA methyltransferase and histone deacetylase restores caspase-8 expression and sensitizes SCLC cells to TRAIL. Carcinogenesis 32, 1450–1458. (doi:10.1093/carcin/bgr135) 21771726 10.1093/carcin/bgr135

[66] Delmonico L et al. 2015 CDKN2A (p14(ARF)/p16(INK4a)) and ATM promoter methylation in patients with impalpable breast lesions. Hum. Pathol. 46, 1540–1547. (doi:10.1016/j.humpath.2015.06.016) 26255234 10.1016/j.humpath.2015.06.016

[67] Verdoodt B, Sommerer F, Palisaar R-J, Noldus J, Vogt M, Nambiar S, Tannapfel A, Mirmohammadsadegh A, Neid M 2011 Inverse association of p16 INK4a and p14 ARF methylation of the CDKN2a locus in different Gleason scores of prostate cancer. Nature 14, 295–301. (doi:10.1038/pcan.2011.45) 10.1038/pcan.2011.45 21912429

[68] Davidović R, Sopta J, Mandušić V, Krajnović M, Stanojević M, Tulić G, Dimitrijević B 2013 p14(ARF) methylation is a common event in the pathogenesis and progression of myxoid and pleomorphic liposarcoma. Med. Oncol. 30, 682 (doi:10.1007/s12032-013-0682-9) 23918242 10.1007/s12032-013-0682-9

[69] Wang Y-J et al. 2014 Epigenetic changes of TIMP-3, GSTP-1 and 14-3-3 sigma genes as indication of status of chronic inflammation and cancer. Int. J. Biol. Markers 29, e208-14 (doi:10.5301/jbm.5000104) 25041782 10.5301/jbm.5000104

[70] Zurita M, Lara PC, del Moral R, Torres B, Linares-Fernández JL, Arrabal SR, Martínez-Galán J, Oliver FJ, de Almodóvar JM Ruiz 2010 Hypermethylated 14-3-3-sigma and ESR1 gene promoters in serum as candidate biomarkers for the diagnosis and treatment efficacy of breast cancer metastasis. BMC Cancer 10, 217 (doi:10.1186/1471-2407-10-217) 20487521 10.1186/1471-2407-10-217 PMC2889892

[71] Yi B et al. 2009 Inactivation of 14-3-3 sigma by promoter methylation correlates with metastasis in nasopharyngeal carcinoma. J. Cell. Biochem. 106, 858–866. (doi:10.1002/jcb.22051) 19160382 10.1002/jcb.22051

[72] Liu L, Yang X 2015 Implication of reprimo and hMLH1 gene methylation in early diagnosis of gastric carcinoma. Int. J. Clin. Exp. Pathol. 8, 14 977–14 982. PMC4713617 26823831

[73] Díaz-Lagares A et al. 2016 Epigenetic inactivation of the p53-induced long noncoding RNA TP53 target 1 in human cancer. Proc. Natl Acad. Sci. USA 113, E7535–E7544. (doi:10.1073/pnas.1608585113) 27821766 10.1073/pnas.1608585113 PMC5127373

[74] Ashkenazi A 2008 Directing cancer cells to self-destruct with pro-apoptotic receptor agonists. Nat. Rev. Drug Discov. 7, 1001–1012. (doi:10.1038/nrd2637) 18989337 10.1038/nrd2637

[75] Alipour M, Zargar SJ, Safarian S, Fouladdel S, Azizi E, Jafargholizadeh N 2013 The study of DNA methylation of bax gene promoter in breast and colorectal carcinoma cell lines. Iran J. Cancer Prev. 6, 59–64. 25250112 PMC4142912

[76] Hervouet E, Cheray M, Vallette FM, Cartron P-F 2013 DNA methylation and apoptosis resistance in cancer cells. Cells 2, 545–573. (doi:10.3390/cells2030545) 24709797 10.3390/cells2030545 PMC3972670

[77] Xiong S 2011 MicroRNA-7 inhibits the growth of human non-small cell lung cancer A549 cells through targeting BCL-2. Int. J. Biol. Sci. 7, 805–814. (doi:10.7150/ijbs.7.805) 21750649 10.7150/ijbs.7.805 PMC3133888

[78] Piazza R et al. 2013 Epigenetic silencing of the proapoptotic gene BIM in anaplastic large cell lymphoma through an MeCP2/SIN3a deacetylating complex. Neoplasia 15, 511–522. (doi:10.1593/neo.121784) 23633923 10.1593/neo.121784 PMC3638354

[79] Zhu X, Yi F, Chen P, Chen L, Zhang X, Cao C, Tan W 2015 5-Aza-2′-deoxycytidine and CDDP synergistically induce apoptosis in renal carcinoma cells via enhancing the APAF-1 activity. Clin. Lab. 61, 1821–1830. (doi:10.7754/Clin.Lab.2015.150429) 26882803 10.7754/clin.lab.2015.150429

[80] Lee KH, Lim SW, Kim HG, Kim DY, Ryu SY, Joo JK, Kim JC, Lee JH 2009 Lack of death receptor 4 (DR4) expression through gene promoter methylation in gastric carcinoma. Langenbecks Arch. Surg. 394, 661–670. (doi:10.1007/s00423-009-0484-x) 19350268 10.1007/s00423-009-0484-x

[81] Skiriute D, Vaitkiene P, Saferis V, Asmoniene V, Skauminas K, Deltuva VP, Tamasauskas A 2012 MGMT, GATA6, CD81, DR4, and CASP8 gene promoter methylation in glioblastoma. BMC Cancer 12, 218 (doi:10.1186/1471-2407-12-218) 22672670 10.1186/1471-2407-12-218 PMC3404983

[82] Butler LM, Dobrovic A, Bianco T, Cowled PA 2000 Promoter region methylation does not account for the frequent loss of expression of the Fas gene in colorectal carcinoma. Br. J. Cancer 82, 131–135. (doi:10.1054/bjoc.1999.0889) 10638979 10.1054/bjoc.1999.0889 PMC2363214

[83] Petak I, Danam RP, Tillman DM, Vernes R, Howell SR, Berczi L, Kopper L, Brent TP, Houghton JA 2003 Hypermethylation of the gene promoter and enhancer region can regulate Fas expression and sensitivity in colon carcinoma. Cell Death Differ. 10, 211–217. (doi:10.1038/sj.cdd.4401132) 12700649 10.1038/sj.cdd.4401132

[84] Watson CJ et al. 2012 Identification of a methylation hotspot in the death receptor Fas/CD95 in bladder cancer. Int. J. Oncol. 40, 645–654. (doi:10.3892/ijo.2011.1250) 22076446 10.3892/ijo.2011.1250

[85] Saberi E, Kordi-Tamandani D-M, Jamali S, Rigi-Ladiz M-A 2014 Analysis of methylation and mRNA expression status of FADD and FAS genes in patients with oral squamous cell carcinoma. Med. Oral Patol. Oral Cir. Bucal. 19, e562–e568. (doi:10.4317/medoral.19805) 25129245 10.4317/medoral.19805 PMC4259371

[86] Terasawa K et al. 2004 Epigenetic inactivation of TMS1/ASC in ovarian cancer. Clin. Cancer Res. 10, 2000–2006. (doi:10.1158/1078-0432.CCR-0932-03) 15041718 10.1158/1078-0432.ccr-0932-03

[87] Stone AR, Bobo W, Brat DJ, Devi NS, Van Meir EG, Vertino PM 2004 Aberrant methylation and down-regulation of TMS1/ASC in human glioblastoma. Am. J. Pathol. 165, 1151–1161. (doi:10.1016/S0002-9440(10)63376-7) 15466382 10.1016/S0002-9440(10)63376-7 PMC1618625

[88] Sakata M et al. 2013 Methylation of the HACE1 gene is frequently detected in hepatocellular carcinoma. Hepatogastroenterology 60, 781–783. (doi:10.5754/hge10439) 23732777 10.5754/hge10439

[89] Koo G-B et al. 2015 Methylation-dependent loss of RIP3 expression in cancer represses programmed necrosis in response to chemotherapeutics. Cell Res. 25, 707–725. (doi:10.1038/cr.2015.56) 25952668 10.1038/cr.2015.56 PMC4456623

[90] Ferreira HJ et al. 2016 DNMT3A mutations mediate the epigenetic reactivation of the leukemogenic factor MEIS1 in acute myeloid leukemia. Oncogene 35, 3079–3082. (doi:10.1038/onc.2015.359) 26434589 10.1038/onc.2015.359 PMC4705435

[91] Ahn J-S et al. 2016 DNMT3A R882 mutation with FLT3-ITD positivity is an extremely poor prognostic factor in patients with normal-karyotype acute myeloid leukemia after allogeneic hematopoietic cell transplantation. Biol. Blood Marrow Transplant. 22, 61–70. (doi:10.1016/j.bbmt.2015.07.030) 26234722 10.1016/j.bbmt.2015.07.030

[92] Liu W-J, Tan X-H, Luo X-P, Guo B-P, Wei Z-J, Ke Q, He S, Cen H 2014 Prognostic significance of Tet methylcytosine dioxygenase 2 (TET2) gene mutations in adult patients with acute myeloid leukemia: a meta-analysis. Leuk. Lymphoma 55, 2691–2698. (doi:10.3109/10428194.2014.893308) 24524305 10.3109/10428194.2014.893308

[93] Eden A, Gaudet F, Waghmare A, Jaenisch R 2003 Chromosomal instability and tumors promoted by DNA hypomethylation. Science 300, 455 (doi:10.1126/science.1083557) 12702868 10.1126/science.1083557

[94] Tufarelli C, Cruickshanks HA, Meehan RR 2013 LINE-1 activation and epigenetic silencing of suppressor genes in cancer: causally related events? Mob. Genet. Elements 3, e26832 (doi:10.4161/mge.26832) 24251074 10.4161/mge.26832 PMC3827066

[95] Cruickshanks HA, Vafadar-Isfahani N, Dunican DS, Lee A, Sproul D, Lund JN, Meehan RR, Tufarelli C 2013 Expression of a large LINE-1-driven antisense RNA is linked to epigenetic silencing of the metastasis suppressor gene TFPI-2 in cancer. Nucleic Acids Res. 41, 6857–6869. (doi:10.1093/nar/gkt438) 23703216 10.1093/nar/gkt438 PMC3737543

[96] Jeggo PA, Pearl LH, Carr AM 2016 DNA repair, genome stability and cancer: a historical perspective. Nat. Rev. Cancer 16, 35–42. (doi:10.1038/nrc.2015.4) 26667849 10.1038/nrc.2015.4

[97] Roos WP, Kaina B 2013 DNA damage-induced cell death: from specific DNA lesions to the DNA damage response and apoptosis. Cancer Lett. 332, 237–248. (doi:10.1016/j.canlet.2012.01.007) 22261329 10.1016/j.canlet.2012.01.007

[98] Gavande NS, VanderVere-Carozza PS, Hinshaw HD, Jalal SI, Sears CR, Pawelczak KS, Turchi JJ 2016 DNA repair targeted therapy: the past or future of cancer treatment? Pharmacol. Ther. 160, 65–83. (doi:10.1016/j.pharmthera.2016.02.003) 26896565 10.1016/j.pharmthera.2016.02.003 PMC4811676

[99] Jackson SP, Bartek J 2009 The DNA-damage response in human biology and disease. Nature 461, 1071–1078. (doi:10.1038/nature08467) 19847258 10.1038/nature08467 PMC2906700

[100] Treilleux I, Chapot B, Goddard S, Pisani P, Angèle S, Hall J 2007 The molecular causes of low ATM protein expression in breast carcinoma; promoter methylation and levels of the catalytic subunit of DNA-dependent protein kinase. Histopathology 51, 63–69. (doi:10.1111/j.1365-2559.2007.02726.x) 17593081 10.1111/j.1365-2559.2007.02726.x

[101] Wang H et al. 2010 Chk2 down-regulation by promoter hypermethylation in human bulk gliomas. Life Sci. 86, 185–191. (doi:10.1016/j.lfs.2009.11.023) 19969004 10.1016/j.lfs.2009.11.023

[102] Sartore-Bianchi A et al. 2017 Digital PCR assessment of MGMT promoter methylation coupled with reduced protein expression optimises prediction of response to alkylating agents in metastatic colorectal cancer patients. Eur. J. Cancer 71, 43–50. (doi:10.1016/j.ejca.2016.10.032) 27997874 10.1016/j.ejca.2016.10.032

[103] Brigliadori G, Foca F, Dall'Agata M, Rengucci C, Melegari E, Cerasoli S, Amadori D, Calistri D, Faedi M 2016 Defining the cutoff value of MGMT gene promoter methylation and its predictive capacity in glioblastoma. J. Neurooncol. 128, 333–339. (doi:10.1007/s11060-016-2116-y) 27029617 10.1007/s11060-016-2116-y

[104] Yoon RG, Kim HS, Paik W, Shim WH, Kim SJ, Kim JH 2017 Different diagnostic values of imaging parameters to predict pseudoprogression in glioblastoma subgroups stratified by MGMT promoter methylation. Eur. Radiol. 27, 255–266. (doi:10.1007/s00330-016-4346-y) 27048531 10.1007/s00330-016-4346-y

[105] Singh KP, Treas J, Tyagi T, Gao W 2012 DNA demethylation by 5-aza-2-deoxycytidine treatment abrogates 17 beta-estradiol-induced cell growth and restores expression of DNA repair genes in human breast cancer cells. Cancer Lett. 316, 62–69. (doi:10.1016/j.canlet.2011.10.022) 22082530 10.1016/j.canlet.2011.10.022

[106] Howard JH et al. 2009 Epigenetic downregulation of the DNA repair gene MED1/MBD4 in colorectal and ovarian cancer. Cancer Biol. Ther. 8, 94–100. (doi:10.4161/cbt.8.1.7469) 19127118 10.4161/cbt.8.1.7469 PMC2683899

[107] Chaisaingmongkol J et al. 2012 Epigenetic screen of human DNA repair genes identifies aberrant promoter methylation of NEIL1 in head and neck squamous cell carcinoma. Oncogene 31, 5108–5116. (doi:10.1038/onc.2011.660) 22286769 10.1038/onc.2011.660

[108] Schärer OD 2013 Nucleotide excision repair in eukaryotes. Cold Spring Harb. Perspect. Biol. 5, a012609 (doi:10.1101/cshperspect.a012609) 24086042 10.1101/cshperspect.a012609 PMC3783044

[109] Wu Y-H, Tsai Chang J-H, Cheng Y-W, Wu T-C, Chen C-Y, Lee H 2007 *Xeroderma pigmentosum* group C gene expression is predominantly regulated by promoter hypermethylation and contributes to p53 mutation in lung cancers. Oncogene 26, 4761–4773. (doi:10.1038/sj.onc.1210284) 17325666 10.1038/sj.onc.1210284

[110] Peng B, Hodge DR, Thomas SB, Cherry JM, Munroe DJ, Pompeia C, Xiao W, Farrar WL 2005 Epigenetic silencing of the human nucleotide excision repair gene, hHR23B, in interleukin-6-responsive multiple myeloma KAS-6/1 cells. J. Biol. Chem. 280, 4182–4187. (doi:10.1074/jbc.M412566200) 15550378 10.1074/jbc.M412566200

[111] Lima LMC, de Souza LR, da Silva TF, Pereira CS, Guimarães ALS, de Paula AMB, de Andrade Carvalho H 2012 DNA repair gene excision repair cross complementing-group 1 (ERCC1) in head and neck squamous cell carcinoma: analysis of methylation and polymorphism (G19007A), protein expression and association with epidemiological and clinicopathological factors. Histopathology 60, 489–496. (doi:10.1111/j.1365-2559.2011.04062.x) 22176134 10.1111/j.1365-2559.2011.04062.x

[112] Muro Y, Sugiura K, Mimori T, Akiyama M 2015 DNA mismatch repair enzymes: genetic defects and autoimmunity. Clin. Chim. Acta 442, 102–109. (doi:10.1016/j.cca.2015.01.014) 25619773 10.1016/j.cca.2015.01.014

[113] Kidambi TD, Blanco A, Van Ziffle J, Terdiman JP 2016 Constitutional MLH1 methylation presenting with colonic polyposis syndrome and not Lynch syndrome. Fam. Cancer 15, 275–280. (doi:10.1007/s10689-016-9868-6) 26781822 10.1007/s10689-016-9868-6

[114] Lee J-H, Paull TT 2004 Direct activation of the ATM protein kinase by the Mre11/Rad50/Nbs1 complex. Science 304, 93–96. (doi:10.1126/science.1091496) 15064416 10.1126/science.1091496

[115] Lord CJ, Ashworth A 2016 BRCAness revisited. Nat. Rev. Cancer 16, 110–120. (doi:10.1038/nrc.2015.21) 26775620 10.1038/nrc.2015.21

[116] Liu S, Li Y, Xu H, Wang K, Li N, Li J, Sun T, Xu Y 2016 Increased expression of SET domain-containing proteins and decreased expression of Rad51 in different classes of renal cell carcinoma. Biosci. Rep. 36, e00349 (doi:10.1042/BSR20160122) 27170370 10.1042/BSR20160122 PMC5293581

[117] Suwaki N, Klare K, Tarsounas M 2011 RAD51 paralogs: roles in DNA damage signalling, recombinational repair and tumorigenesis. Semin. Cell Dev. Biol. 22, 898–905. (doi:10.1016/j.semcdb.2011.07.019) 21821141 10.1016/j.semcdb.2011.07.019

[118] Rieke DT, Ochsenreither S, Klinghammer K, Seiwert TY, Klauschen F, Tinhofer I, Keilholz U 2016 Methylation of RAD51B, XRCC3 and other homologous recombination genes is associated with expression of immune checkpoints and an inflammatory signature in squamous cell carcinoma of the head and neck, lung and cervix. Oncotarget 7, 75 379–75 393. (doi:10.18632/oncotarget.12211) 10.18632/oncotarget.12211 PMC5342748 27683114

[119] Pathania S, Nguyen J, Hill SJ, Scully R, Adelmant GO, Marto JA, Feunteun J, Livingston DM 2011 BRCA1 is required for postreplication repair after UV-induced DNA damage. Mol. Cell 44, 235–251. (doi:10.1016/j.molcel.2011.09.002) 21963239 10.1016/j.molcel.2011.09.002 PMC3200447

[120] O'Driscoll M, Jeggo PA 2006 The role of double-strand break repair—insights from human genetics. Nat. Rev. Genet. 7, 45–54. (doi:10.1038/nrg1746) 16369571 10.1038/nrg1746

[121] Moutinho C et al. 2014 Epigenetic inactivation of the BRCA1 interactor SRBC and resistance to oxaliplatin in colorectal cancer. J. Natl Cancer Inst. 106, djt322 (doi:10.1093/jnci/djt322) 24273214 10.1093/jnci/djt322 PMC3906989

[122] Masuda K et al. 2012 Association of epigenetic inactivation of the WRN gene with anticancer drug sensitivity in cervical cancer cells. Oncol. Rep. 28, 1146–1152. (doi:10.3892/or.2012.1912) 22797812 10.3892/or.2012.1912 PMC3583574

[123] Nogales V et al. 2016 Epigenetic inactivation of the putative DNA/RNA helicase SLFN11 in human cancer confers resistance to platinum drugs. Oncotarget 7, 3084–3097. (doi:10.18632/oncotarget.6413) 26625211 10.18632/oncotarget.6413 PMC4823092

[124] Mu Y, Lou J, Srivastava M, Zhao B, Feng X-H, Liu T, Chen J, Huang J 2016 SLFN11 inhibits checkpoint maintenance and homologous recombination repair. EMBO Rep. 17, 94–109. (doi:10.15252/embr.201540964) 26658330 10.15252/embr.201540964 PMC4718411

[125] Zimmermann M, de Lange T 2014 53BP1: pro choice in DNA repair. Trends Cell Biol. 24, 108–117. (doi:10.1016/j.tcb.2013.09.003) 24094932 10.1016/j.tcb.2013.09.003 PMC3946699

[126] Davis AJ, Chen DJ 2013 DNA double strand break repair via non-homologous end-joining. Transl. Cancer Res. 2, 130–143. (doi:10.3978/j.issn.2218-676X.2013.04.02) 24000320 10.3978/j.issn.2218-676X.2013.04.02 PMC3758668

[127] Zhou C, Tang H, Yu J, Zhuang D, Zhang H 2015 Blood-based DNA methylation of DNA repair genes in the non-homologous end-joining (NEHJ) pathway in patient with glioma. Int. J. Clin. Exp. Pathol. 8, 9463–9467. 26464705 PMC4583937

[128] Carmona FJ et al. 2014 A comprehensive DNA methylation profile of epithelial-to-mesenchymal transition. Cancer Res. 74, 5608–5619. (doi:10.1158/0008-5472.CAN-13-3659) 25106427 10.1158/0008-5472.CAN-13-3659

[129] Yang M-H, Chen C-L, Chau G-Y, Chiou S-H, Su C-W, Chou T-Y, Peng W-L, Wu J-C 2009 Comprehensive analysis of the independent effect of twist and snail in promoting metastasis of hepatocellular carcinoma. Hepatology 50, 1464–1474. (doi:10.1002/hep.23221) 19821482 10.1002/hep.23221

[130] Li N, Li S 2015 Epigenetic inactivation of SOX1 promotes cell migration in lung cancer. Tumour Biol. 36, 4603–4610. (doi:10.1007/s13277-015-3107-x) 25613070 10.1007/s13277-015-3107-x

[131] Li H et al. 2014 Epigenetic inactivation of KLF4 is associated with urothelial cancer progression and early recurrence. J. Urol. 191, 493–501. (doi:10.1016/j.juro.2013.08.087) 24018236 10.1016/j.juro.2013.08.087

[132] Li P, Liu X, Dong Z-M, Ling Z-Q 2015 Epigenetic silencing of HIC1 promotes epithelial-mesenchymal transition and drives progression in esophageal squamous cell carcinoma. Oncotarget 6, 38 151–38 165. (doi:10.18632/oncotarget.5832) 10.18632/oncotarget.5832 PMC4741990 26510908

[133] Yan W, Wu K, Herman JG, Brock MV, Zhou Y, Lu Y, Zhang Z, Yang Y, Guo M 2014 Epigenetic silencing of DACH1 induces the invasion and metastasis of gastric cancer by activating TGF-β signalling. J. Cell. Mol. Med. 18, 2499–2511. (doi:10.1111/jcmm.12325) 24912879 10.1111/jcmm.12325 PMC4302654

[134] Mustafa M, Lee J-Y, Kim MH 2015 CTCF negatively regulates HOXA10 expression in breast cancer cells. Biochem. Biophys. Res. Commun. 467, 828–834. (doi:10.1016/j.bbrc.2015.10.058) 26478432 10.1016/j.bbrc.2015.10.058

[135] Fang Z, Yao W, Xiong Y, Zhang J, Liu L, Li J, Zhang C, Wan J 2010 Functional elucidation and methylation-mediated downregulation of ITGA5 gene in breast cancer cell line MDA-MB-468. J. Cell. Biochem. 110, 1130–1141. (doi:10.1002/jcb.22626) 20564209 10.1002/jcb.22626

[136] Lin Y-L, Gui S-L, Ma J-G 2015 Aberrant methylation of CDH11 predicts a poor outcome for patients with bladder cancer. Oncol. Lett. 10, 647–652. (doi:10.3892/ol.2015.3337) 26622548 10.3892/ol.2015.3337 PMC4509141

[137] van Baars R et al. 2016 CADM1 and MAL methylation status in cervical scrapes is representative of the most severe underlying lesion in women with multiple cervical biopsies. Int. J. Cancer 138, 463–471. (doi:10.1002/ijc.29706) 26219541 10.1002/ijc.29706

[138] Guo L-L, He Z-C, Yang C-Q, Qiao P-T, Yin G-L 2015 Epigenetic silencing of olfactomedin-4 enhances gastric cancer cell invasion via activation of focal adhesion kinase signaling. BMB Rep. 48, 630–635. (doi:10.5483/BMBRep.2015.48.11.130) 26303970 10.5483/BMBRep.2015.48.11.130 PMC4911205

[139] Lu C-H, Wu W-Y, Lai Y-J, Yang C-M, Yu L-C 2014 Suppression of B3GNT7 gene expression in colon adenocarcinoma and its potential effect in the metastasis of colon cancer cells. Glycobiology 24, 359–367. (doi:10.1093/glycob/cwu002) 24418929 10.1093/glycob/cwu002

[140] Ding W, Fan X-L, Xu X, Huang J-Z, Xu S-H, Geng Q, Li R, Chen D, Yan G-R 2015 Epigenetic silencing of ITGA2 by MiR-373 promotes cell migration in breast cancer. PLoS ONE 10, e0135128 (doi:10.1371/journal.pone.0135128) 26258411 10.1371/journal.pone.0135128 PMC4530956

[141] Seol HS, Akiyama Y, Shimada S, Lee HJ, Kim TI, Chun SM, Singh SR, Jang SJ 2014 Epigenetic silencing of microRNA-373 to epithelial-mesenchymal transition in non-small cell lung cancer through IRAK2 and LAMP1 axes. Cancer Lett. 353, 232–241. (doi:10.1016/j.canlet.2014.07.019) 25063738 10.1016/j.canlet.2014.07.019 PMC7707239

[142] Korpal M, Lee ES, Hu G, Kang Y 2008 The miR-200 family inhibits epithelial-mesenchymal transition and cancer cell migration by direct targeting of E-cadherin transcriptional repressors ZEB1 and ZEB2. J. Biol. Chem. 283, 14 910–14 914. (doi:10.1074/jbc.C800074200) 10.1074/jbc.C800074200 PMC3258899 18411277

[143] Planagumà J., Liljeström M, Alameda F, Bützow R, Virtanen I, Reventós J, Hukkanen M 2011 Matrix metalloproteinase-2 and matrix metalloproteinase-9 codistribute with transcription factors RUNX1/AML1 and ETV5/ERM at the invasive front of endometrial and ovarian carcinoma. Hum. Pathol. 42, 57–67. (doi:10.1016/j.humpath.2010.01.025) 20970160 10.1016/j.humpath.2010.01.025

[144] Dong W, Chen X, Xie J, Sun P, Wu Y 2010 Epigenetic inactivation and tumor suppressor activity of HAI-2/SPINT2 in gastric cancer. Int. J. Cancer 127, 1526–1534. (doi:10.1002/ijc.25161) 20063316 10.1002/ijc.25161

[145] Yue D et al. 2014 Epigenetic inactivation of SPINT2 is associated with tumor suppressive function in esophageal squamous cell carcinoma. Exp. Cell Res. 322, 149–158. (doi:10.1016/j.yexcr.2013.11.009) 24269829 10.1016/j.yexcr.2013.11.009

[146] Hwang S, Kim H-E, Min M, Raghunathan R, Panova IP, Munshi R, Ryu B 2015 Epigenetic silencing of SPINT2 promotes cancer cell motility via HGF-MET pathway activation in melanoma. J. Invest. Dermatol. 135, 2283–2291. (doi:10.1038/jid.2015.160) 25910030 10.1038/jid.2015.160 PMC4537358

[147] Wang L, Ge J, Ma T, Zheng Y, Lv S, Li Y, Liu S 2015 Promoter hypermethylation of the cysteine protease RECK may cause metastasis of osteosarcoma. Tumour Biol. 36, 9511–9516. (doi:10.1007/s13277-015-3688-4) 26130413 10.1007/s13277-015-3688-4

[148] Zhang Y, Huang Z, Zhu Z, Zheng X, Liu J, Han Z, Ma X, Zhang Y 2014 Upregulated UHRF1 promotes bladder cancer cell invasion by epigenetic silencing of KiSS1. PLoS ONE 9, e104252 (doi:10.1371/journal.pone.0104252) 25272010 10.1371/journal.pone.0104252 PMC4182677

[149] Wu D, Li M, Wang L, Zhou Y, Zhou J, Pan H, Qu P 2014 microRNA-145 inhibits cell proliferation, migration and invasion by targeting matrix metallopeptidase-11 in renal cell carcinoma. Mol. Med. Rep. 10, 393–398. (doi:10.3892/mmr.2014.2149) 24737449 10.3892/mmr.2014.2149

[150] Zhou H, Huang S 2011 Role of mTOR signaling in tumor cell motility, invasion and metastasis. Curr. Protein Pept. Sci. 12, 30–42. (doi:10.2174/138920311795659407) 21190521 10.2174/138920311795659407 PMC3410744

[151] Wei F-Z et al. 2015 Epigenetic regulation of autophagy by the methyltransferase EZH2 through an MTOR-dependent pathway. Autophagy 11, 2309–2322. (doi:10.1080/15548627.2015.1117734) 26735435 10.1080/15548627.2015.1117734 PMC4835210

[152] Chen L, Li X, Chen X 2015 Prognostic significance of tissue miR-345 downregulation in non-small cell lung cancer. Int. J. Clin. Exp. Med. 8, 20 971–20 976. 26885027 PMC4723872

[153] Yu M et al. 2017 miR-345 inhibits tumor metastasis and EMT by targeting IRF1-mediated mTOR/STAT3/AKT pathway in hepatocellular carcinoma. Int. J. Oncol. 50, 975–983. (doi:10.3892/ijo.2017.3852) 28098858 10.3892/ijo.2017.3852

[154] Xu J et al. 2012 Epigenetic regulation of HIF-1α in renal cancer cells involves HIF-1α/2α binding to a reverse hypoxia-response element. Oncogene 31, 1065–1072. (doi:10.1038/onc.2011.305) 21841824 10.1038/onc.2011.305

[155] Vander Heiden MG et al. 2011 Metabolic pathway alterations that support cell proliferation. Cold Spring Harb. Symp. Quant. Biol. 76, 325–334. (doi:10.1101/sqb.2012.76.010900) 22262476 10.1101/sqb.2012.76.010900

[156] Liu VM, Vander Heiden MG 2015 The role of pyruvate kinase M2 in cancer metabolism. Brain Pathol. 25, 781–783. (doi:10.1111/bpa.12311) 26526946 10.1111/bpa.12311 PMC8029046

[157] Wamelink MMC, Struys EA, Jakobs C 2008 The biochemistry, metabolism and inherited defects of the pentose phosphate pathway: a review. J. Inherit. Metab. Dis. 31, 703–717. (doi:10.1007/s10545-008-1015-6) 18987987 10.1007/s10545-008-1015-6

[158] Lopez-Serra P et al. 2014 A DERL3-associated defect in the degradation of SLC2A1 mediates the Warburg effect. Nat. Commun. 5, 3608 (doi:10.1038/ncomms4608) 24699711 10.1038/ncomms4608 PMC3988805

[159] Mondesir J, Willekens C, Touat M, de Botton S 2016 IDH1 and IDH2 mutations as novel therapeutic targets: current perspectives. J. Blood Med. 7, 171–180. (doi:10.2147/JBM.S70716) 27621679 10.2147/JBM.S70716 PMC5015873

[160] Parker SJ, Metallo CM 2015 Metabolic consequences of oncogenic IDH mutations. Pharmacol. Ther. 152, 54–62. (doi:10.1016/j.pharmthera.2015.05.003) 25956465 10.1016/j.pharmthera.2015.05.003 PMC4489982

[161] Viswanath P, Chaumeil MM, Ronen SM 2016 Molecular imaging of metabolic reprograming in mutant IDH cells. Front. Oncol. 6, 60 (doi:10.3389/fonc.2016.00060) 27014635 10.3389/fonc.2016.00060 PMC4789800

[162] Ohba S et al. 2016 Mutant IDH1 expression drives TERT promoter reactivation as part of the cellular transformation process. Cancer Res. 76, 6680–6689. (doi:10.1158/0008-5472.CAN-16-0696) 27758882 10.1158/0008-5472.CAN-16-0696 PMC5290072

[163] Baggott JE, Tamura T 2015 Folate-dependent purine nucleotide biosynthesis in humans. Adv. Nutr. 6, 564–571. (doi:10.3945/an.115.008300) 26374178 10.3945/an.115.008300 PMC4561830

[164] Gao X, Reid MA, Kong M, Locasale JW 2017 Metabolic interactions with cancer epigenetics. Mol. Aspects Med. 55, 20–25. (doi:10.1016/j.mam.2016.09.001) 27620316 10.1016/j.mam.2016.09.001 PMC5344790

[165] Zhao H et al. 2012 Frequent epigenetic silencing of the folate-metabolising gene cystathionine-beta-synthase in gastrointestinal cancer. PLoS ONE 7, e49683 (doi:10.1371/journal.pone.0049683) 23152928 10.1371/journal.pone.0049683 PMC3496708

[166] Jeschke J et al. 2013 Frequent inactivation of cysteine dioxygenase type 1 contributes to survival of breast cancer cells and resistance to anthracyclines. Clin. Cancer Res. 19, 3201–3211. (doi:10.1158/1078-0432.CCR-12-3751) 23630167 10.1158/1078-0432.CCR-12-3751 PMC3985391

[167] Archer SL 2016 Acquired mitochondrial abnormalities, including epigenetic inhibition of superoxide dismutase 2, in pulmonary hypertension and cancer: therapeutic implications. Adv. Exp. Med. Biol. 903, 29–53. (doi:10.1007/978-1-4899-7678-9_3) 27343087 10.1007/978-1-4899-7678-9_3

[168] Liu Q, Jin J, Ying J, Sun M, Cui Y, Zhang L, Xu B, Fan Y, Zhang Q 2015 Frequent epigenetic suppression of tumor suppressor gene glutathione peroxidase 3 by promoter hypermethylation and its clinical implication in clear cell renal cell carcinoma. Int. J. Mol. Sci. 16, 10 636–10 649. (doi:10.3390/ijms160510636) 10.3390/ijms160510636 PMC4463666 25970749

[169] Damhofer H, Medema JP, Veenstra VL, Badea L, Popescu I, Roelink H, Bijlsma MF 2013 Assessment of the stromal contribution to Sonic Hedgehog-dependent pancreatic adenocarcinoma. Mol. Oncol. 7, 1031–1042. (doi:10.1016/j.molonc.2013.08.004) 23998958 10.1016/j.molonc.2013.08.004 PMC3838447

[170] Tang S-N, Fu J, Nall D, Rodova M, Shankar S, Srivastava RK 2012 Inhibition of sonic hedgehog pathway and pluripotency maintaining factors regulate human pancreatic cancer stem cell characteristics. Int. J. Cancer 131, 30–40. (doi:10.1002/ijc.26323) 21796625 10.1002/ijc.26323 PMC3480310

[171] Heiden KB, Williamson AJ, Doscas ME, Ye J, Wang Y, Liu D, Xing M, Prinz RA, Xu X 2014 The sonic hedgehog signaling pathway maintains the cancer stem cell self-renewal of anaplastic thyroid cancer by inducing snail expression. J. Clin. Endocrinol. Metab. 99, E2178–E2187. (doi:10.1210/jc.2014-1844) 25078145 10.1210/jc.2014-1844 PMC5393503

[172] Schmidt-Wolf IGH, Plass C, Byrd JC, Frevel K, Pietsch T, Waha A 2016 Assessment of promoter methylation identifies PTCH as a putative tumor-suppressor gene in human CLL. Anticancer Res. 36, 4515–4519. (doi:10.21873/anticanres.10998) 27630290 10.21873/anticanres.10998

[173] Chen J-S et al. 2014 Down-regulation of Gli-1 inhibits hepatocellular carcinoma cell migration and invasion. Mol. Cell. Biochem. 393, 283–291. (doi:10.1007/s11010-014-2071-x) 24792036 10.1007/s11010-014-2071-x

[174] Singh I et al. 2014 Hmga2 is required for canonical WNT signaling during lung development. BMC Biol. 12, 21 (doi:10.1186/1741-7007-12-21) 24661562 10.1186/1741-7007-12-21 PMC4064517

[175] Morishita A, Zaidi MR, Mitoro A, Sankarasharma D, Szabolcs M, Okada Y, D'Armiento J, Chada K 2013 HMGA2 is a driver of tumor metastasis. Cancer Res. 73, 4289–4299. (doi:10.1158/0008-5472.CAN-12-3848) 23722545 10.1158/0008-5472.CAN-12-3848 PMC3715567

[176] Mayr C, Hemann MT, Bartel DP 2007 Disrupting the pairing between let-7 and Hmga2 enhances oncogenic transformation. Science 315, 1576–1579. (doi:10.1126/science.1137999) 17322030 10.1126/science.1137999 PMC2556962

[177] Aithal MG. S, Rajeswari N 2013 Role of Notch signalling pathway in cancer and its association with DNA methylation. J. Genet. 92, 667–675. (doi:10.1007/s12041-013-0284-5) 24371188 10.1007/s12041-013-0284-5

[178] Karon M, Sieger L, Leimbrock S, Finklestein JZ, Nesbit ME, Swaney JJ 1973 5-Azacytidine: a new active agent for the treatment of acute leukemia. Blood 42, 359–365. 4125239

[179] Ritchie EK, Feldman EJ, Christos PJ, Rohan SD, Lagassa CB, Ippoliti C, Scandura JM, Carlson K, Roboz GJ 2013 Decitabine in patients with newly diagnosed and relapsed acute myeloid leukemia. Leuk. Lymphoma 54, 2003–2007. (doi:10.3109/10428194.2012.762093) 23270581 10.3109/10428194.2012.762093 PMC3888021

[180] Damaskos C et al. 2016 Histone deacetylase inhibitors: a novel therapeutic weapon against medullary thyroid cancer? Anticancer Res. 36, 5019–5024. (doi:10.21873/anticanres.11070) 27798860 10.21873/anticanres.11070

[181] Brown JAL, Bourke E, Eriksson LA, Kerin MJ 2016 Targeting cancer using KAT inhibitors to mimic lethal knockouts. Biochem. Soc. Trans. 44, 979–986. (doi:10.1042/BST20160081) 27528742 10.1042/BST20160081 PMC4984449

[182] Song Y, Wu F, Wu J 2016 Targeting histone methylation for cancer therapy: enzymes, inhibitors, biological activity and perspectives. J. Hematol. Oncol. 9, 49 (doi:10.1186/s13045-016-0279-9) 27316347 10.1186/s13045-016-0279-9 PMC4912745

[183] Pérez-Salvia M, Esteller M 2016 Bromodomain inhibitors and cancer therapy: from structures to applications. Epigenetics 12, 323–339. (doi:10.1080/15592294.2016.1265710) 27911230 10.1080/15592294.2016.1265710 PMC5453193

[184] Enríquez P 2016 CRISPR-mediated epigenome editing. Yale J. Biol. Med. 89, 471–486. 28018139 PMC5168826

[185] Delpu Y, Cordelier P, Cho WC, Torrisani J 2013 DNA methylation and cancer diagnosis. Int. J. Mol. Sci. 14, 15 029–15 058. (doi:10.3390/ijms140715029) 10.3390/ijms140715029 PMC3742286 23873296

[186] Wang J, Han X, Sun Y 2017 DNA methylation signatures in circulating cell-free DNA as biomarkers for the early detection of cancer. Sci. China Life Sci. 60, 356–362. (doi:10.1007/s11427-016-0253-7) 28063009 10.1007/s11427-016-0253-7

[187] Morán S et al. 2016 Epigenetic profiling to classify cancer of unknown primary: a multicentre, retrospective analysis. Lancet Oncol. 17, 1386–1395. (doi:10.1016/S1470-2045(16)30297-2) 27575023 10.1016/S1470-2045(16)30297-2

[188] Vizoso M et al. 2015 Epigenetic activation of a cryptic TBC1D16 transcript enhances melanoma progression by targeting EGFR. Nat. Med. 21, 741–750. (doi:10.1038/nm.3863) 26030178 10.1038/nm.3863 PMC4968631

[189] Esteller M, Garcia-Foncillas J, Andion E, Goodman SN, Hidalgo OF, Vanaclocha V, Baylin SB, Herman JG 2000 Inactivation of the DNA-repair gene MGMT and the clinical response of gliomas to alkylating agents. N. Engl. J. Med. 343, 1350–1354. (doi:10.1056/NEJM200011093431901) 11070098 10.1056/NEJM200011093431901

[190] Sun-tzu. 2008 The art of war. London, UK: Penguin Books Ltd.

